# Clinical Review and Hospital Reports

**Published:** 1834-07-01

**Authors:** 


### Clinical Review.

### Some Remarks on the Venereal Condyloma and on Venereal Warts. By Henry James Johnson, late House-Surgeon to the Lock-Hospital.

The venereal condyloma does not appear to me to have received so much attention as it merits. It is commonly considered to be merely the result of acrimonious secretions, or of want of cleanliness: and personal attention, with simple remedies, are usually considered, sufficient for its cure. When it does not yield to means of this description, escharotics are frequently applied with freedom, but many practitioners appear, if I may venture to make the observation, to be destitute of definite ideas of its nature, and of any appropriate system of management.

It is my object in this paper to lay before my readers, in as clear and concise a manner as possible, the most important facts connected with this subject—to endeavour, from the phenomena, to determine the nature of the venereal condyloma—to distinguish its varieties, if such exist—and to aim at founding, on something like determinate arrangement, a definite, or, at least, consistent mode of treatment. There are numerous difficulties in the path of the inquirer, and perhaps I may hope to stimulate others, possessed of greater powers of analysis, to effect their removal more successfully than myself.

It is a rule, I believe, in logic, that disputants agree in the definition of the name and nature of their subject. The neglect of this precaution has occasioned much dispute in the world of medicine. It is therefore necessary to acquaint my readers with what they are to understand by the term venereal condyloma. I mean by it an interstitial thickening of the cutis, more or less disposed to circularity, smooth, or, at least, not fissured nor lobulated like warts upon the surface, and possessed of considerable vascular organization. The distinctions between this and wart are apparent, but they glide into each other, and instances occur in which it is not easy to assign the preponderating character.

It may not be out of place to exhibit the imperfections of the common descriptions of venereal condyloma. Mr. Cooper's Surgical Dictionary, which is generally admitted to contain an epitome of the actual state of surgical knowledge, disposes of this affection in a summary manner. The whole of the article is comprised in three sentences. "Condyloma," says Mr. Cooper, "is a small, very hard tumor. The term is generally applied to excrescences of this description about the anus. The practitioner may either destroy them with caustic, tie their base with a ligature, or remove them at once with a knife: the first is generally the worst; the last, the best and most speedy method."—*Sixth edition*, 1830.

It will probably appear, that brief as the foregoing description is, inadequate as it must be to portray an affection so frequent and so singular, it is long enough to contain some serious errors. No reference is made to the complaint in the work of Dr. Titley, which purports to treat of the venereal disease—nor in that of Mr. Carmichael—nor in others to which it is unnecessary to allude. It is sufficient to observe that, so far as I know, there is no good description of the condyloma, and that what I am about to offer is exclusively drawn from personal observation. This must be received as an apology for its defects.

The venereal condyloma, or that condition I have endeavoured to define, appears under three varieties of form. It is probable, however, that they are rather degrees of the same complaint, than strict and separate varieties. The division is necessary for convenience of description and methodical arrangement, but the practical surgeon disregards at the bed-side these artificial separations of phenomena naturally connected with each other.

The most simple form of condyloma is a flat, superficial, nearly circular deposit in the cutis. It is soft, and smooth upon the surface—it varies in thickness from that of a sixpenny to that of a penny-piece—in circumference it seldom exceeds the former, and is often little larger than a split-pea—it is evidently vascular—and its tint is usually redder than that of the surrounding skin.

Like the other varieties, it is much more frequent in women than in men, indeed I have seen few examples of it in the latter.

Its situation in both is that which is most favourable to the production and maintenance of heat and moisture, and to the lodgment of discharge from the urethra or vagina. In females, for instance, the lower half of the labia, especially their internal surface—the perineum—the nates where opposed to each other, and contiguous to the anus—the thighs where they join the perineum, are the parts where condylomata luxuriate. In the male, the lower surface of the penis, where it hangs against the scrotum—and the sides of the latter, where they meet the thighs, are the usual situations for their growth. A male out-patient of the hospital had so numerous an array of these formations that his genital organs wore a singular appearance; the scrotum presented a ridiculous resemblance to those antique shields, which are closely studded with flat circular bosses.

In either sex this form of condyloma is accompanied with discharge from the urethra or vagina. It is more than probable that it never occurs except as a consequence of such discharge. The circumstances, the habits, and the conformation of the female render her more liable to profuse secretion from the genital organs than the male. In the lower class of women that infest the streets, and form the majority of the inmates of the hospital, this form of condyloma is extremely frequent. The accounts which they give of their complaints can seldom be depended on, yet they usually admit that discharge preceded the appearance of the condyloma. Yet the latter would seem to occur more readily in some individuals than in others. It may be presumed that in such instances the secretions are peculiarly acrid.

The discharge that attends condyloma is commonly yellowish, profuse, and thin. It is, in fact, chronic gonorrhœa, and the more severe symptoms of that complaint are comparatively rarely present.

Perhaps it would be better to dispose of this, before I proceed to the other varieties. The treatment is the only point which requires farther notice. It consists of two items—prevention and cure: prevention of all that excites the morbid growth—removal of that which has been formed.

The preventive treatment is simple and obvious. Cleanliness—the regular use of the hip-bath—a mild, unirritating diet, comprising little animal food, and no stimulating fluids—aperients—astringent injections thrown into the vagina or urethra. A remark may be made with respect to aperients and injections. It is usually better to commence the former with some doses of calomel or blue pill. This may be given every night for a few times, and may then be continued on alternate nights for a week or ten days: a draught containing senna and the sulphate of magnesia being taken on the following mornings. Afterwards, aperients, not containing mercury, must be steadily exhibited. The combination of rhubarb and magnesia, or that of the sulphate of the latter with the carbonate generally answers very well.

I think that astringent injections are preferable to those of a stimulating nature. The injection of lead and decoction of poppies, or of alum and decoction of oak bark, is commonly more effectual than that of the sulphate of zinc, of the oxymuriate of mercury, or even of the nitrate of silver, much as the latter has been lauded.*

* The reader may refer to two papers on Gonorrhœa, published in the

The removal of the condylomatous deposites that are formed is a matter of importance, and, at times, of difficulty. It is singular that they yield much more readily in some cases than in others. Excision is almost impracticable, certainly improper — the ligature cannot be applied—powerful escharotics are usually unnecessary, and therefore, from the pain they occasion, reprehensible. Sometimes the steady use of cloths dipped in powerful saturnine lotions is sufficient, when combined with the remedies already pointed out. At other times this will not succeed. In most cases the lotion of the oxymuriate of mercury answers remarkably well. It forms the staple application of the hospital. At first it may be used of the strength of half a grain of the oxymuriate to the ounce of distilled water ; the proportion of the former being gradually augmented, according to circumstances, to two grains, or even to three. The condylomata may be bathed with this wash twice or thrice in the day, and lint dipped in it should be constantly applied to them. I should state that I have seen this occasion salivation.

The second variety of condylomatous affection would appear to be merely an advanced degree of the former, and yet there are attendant circumstances which render both it and the succeeding peculiarly worthy of attention. They constitute a frequent form of venereal sore, and are often followed by secondary symptoms. I suspect that the descriptions of the elevated sore, contained in the works of some writers on venereal diseases, are partially derived from the kind of condyloma to which I shall now draw attention.

The second variety of condyloma is distinguished from the first by the fact of ulceration having taken place upon the surface. Each particular condylomatous deposit is a sore. On some, the ulceration is very superficial, the surface resembling mucous membrane, or very fine cuticle, in appearance; on others it is distinct flat ulceration ; and on others, again, the ulceration may be rather cupped. The tint is generally red, seldom, except in the cupped ulcerations, yellow. The base, or substratum, of all is firm, for the obvious reason that each is a condylomatous deposite. For the same reason all the ulcerations, or sores, are raised above the level of the neighbouring integuments, all being, therefore, more or less elevated sores. The elevation depends on the existence of the condyloma, not on changes dependent on the sore itself, a distinctive fact, which should not be forgotten.

The essential features of these condylomatous sores may be briefly recapitulated—their number, size, and situation following the laws alluded to in the description of the simple condyloma—their elevated character—the flatness of their surface.

These condylomatous sores at times affect a form which is rather peculiar. A row of ulcerations may be seen on the free edge of either labium, extending from the upper to the lower commissure, corresponding upon either side, and looking not unlike a row of beads. This form of sore is usually small, not exceeding, perhaps, hardly equalling in size a silver twopenny-piece.

The ulcerated condyloma is palpably possessed of high contagious properties. Opposite surfaces infect each other, and wherever the discharge descends, fresh sores will usually spring up. I have seen them, in uncleanly women, stretch in scattered order, down the inside of the thigh to the knee. The bead-like sores upon the labia are evidently dependent on the power of infection possessed by the discharge.

The ulcerated condyloma, like the simple form, is much more frequent in women than in men. Probably, the majority of the women received into the hospital display this kind of ulceration.

It is usually attended with profuse vaginal or urethral discharge. I was tempted, in many instances, to examine the interior of the vagina with the speculum. I must own that, with the ex-

---

38th and 39th Numbers of this Journal. They contain the author's views upon this subject, although they require rearrangement.

ception of redness of the mucous membrane, and in a few instances, something like abrasion, I found no appearances to satisfy my curiosity.

Such is the description, imperfect it is true, which my observations enable me to offer. If it leads other surgeons to study the affection with more care, and to discriminate its characters with more success, all I can venture to expect will be obtained.

It remains for me to advert to those secondary consequences of the ulcerated condyloma, which entitle it to rank amongst the forms of syphilitic sore.

Those secondary consequences consist of bubo, ulceration of the tonsils, a peculiar affection of the lip and of the tongue, and stains or scaly eruptions on the skin.

Bubo is seldom noticed in the simple form of condyloma; but as that is an effect of chronic discharge, and as enlargements of the inguinal glands, from irritation, may at any time accompany such a condition it cannot be a matter of surprize if bubo does at times occur. In the simple condyloma bubo very seldom suppurates, at least, I have rarely observed it do so. Yet the suppuration of a bubo depends so much on the conduct of the patient and the treatment of the surgeon, that no important inferences can be founded upon it.

In the ulcerated condyloma, bubo is more frequent, and apparently, more disposed to suppuration. Yet the latter event is uncommon, even in the class of persons received into the hospital, a class in whom every circumstance is present which can aggravate venereal symptoms.

The ulcerated form of condyloma has seldom existed for any length of time, without the occurrence of ulceration of the tonsils. This is usually of a well-marked character. The tonsils are commonly enlarged, sometimes very much so. Their surface displays a peculiar whitish ulceration. For the most part this is superficial; I have never seen it deep. Sometimes the ulceration is of a yellowish tint, with little enlargement of the tonsils, but the former state is by far the most frequent. Indeed the experienced surgeon may in numerous instances predict this state of tonsils from observing the condition of the condyloma. I would wish to draw particular attention to these circumstances, as the combination of white ulceration of the tonsils with condyloma has never, so far as I know, been pointed out. Indeed, I am not aware that any of the secondary symptoms from condyloma have been noticed.

Sometimes the soft palate displays the same kind of ulceration as the tonsils. It is generally free from it, and merely exhibits an increase of vascularity.

The eruption on the skin consists of the mere stain, or of spots of a scaly character. The latter may assume the form of the lepra, the psoriasis, or the desquamating tubercle. The secondary syphilitic eruptions will be noticed more fully in a future paper. My remarks at present will be very brief.

The eruptions in question appear to admit of a natural arrangement into three leading classes—the papular eruptions, which evince a disposition to the formation of small vesicles or pustules at the summits of the papulæ—the pustular eruptions, on the one hand, of only small pustules, which approach to papulæ in character, and, on the other, comprising rupia, and ecthyma—and the scaly eruptions, with which must be ranked the pityriasis-like stain.

An able and experienced modern author, Mr. Carmichael, of Dublin, has attempted to prove that certain eruptions generally follow certain sores. On another occasion I may offer some reasons for dissenting from this doctrine. At present it may perhaps be sufficient to observe, that certain eruptions predominate in certain states of constitution. This is not the expression of the whole truth, but the state of constitution is extremely operative in determining the kind of eruption, as well as the nature of the ulceration of the throat.

It would exceed my limits and be inconsistent with my plan to go into this subject more deeply. I must therefore return to the primary statement, that in cases of ulcerated condyloma the patient is frequently affected with eruptions on the skin, consisting of mere stain—of lepra—of psoriasis—or of desquamating tubercle. A few words on each may probably be permitted.

The stain is usually of a yellowish, sometimes of a purplish brown tint. It is of variable intensity, being sometimes very deep in colour, sometimes almost imperceptible. It consists of patches of irregular form, and of small, though indefinite, size. They are usually clustered. They are most apparent on the lower part of the abdomen, the breast, and the back part of the neck. The cuticle is slightly disposed to scale. There is no appreciable deposite in the cutis.

Of the lepra I need not speak, as Bateman has drawn it in his plates. In lepra there is a manifest deposite in the cutis, and the cuticle desquamates upon it.

The psoriasis, I must be pardoned for employing the term, as a better fails to present itself, consists of small, separate deposites in the cutis. They approach in size to split peas, are sometimes confluent, of brownish red colour, flat, and desquamating on the surface.

The tubercle is formed of a more round and bulky deposite than the former. It feels like a pea in the cutis. Its tint is commonly more florid. It desquamates upon the surface.

The analogy between these four forms of eruption is shewn by two circumstances; first, by their frequent coexistence, the same patient displaying two or even all at the same time; secondly, by their succession, one very commonly following the other.

These eruptions are not in themselves an evidence of a bad condition of the system. *That* is marked by the pustules which end in ulceration and in crust. In these eruptions, the tendency is to deposite, rather than to ulceration, an important feature. Previous to quitting this subject I may mention, that in all eruptions the patches are usually larger, and the disposition to crust is greater in the lower extremities, than in the trunk or in the arms.

I stated that a peculiar affection of the lip and of the tongue must be ranked among the secondary symptoms of condyloma. I have only seen one instance of each.

The affection of the lip occurred in a girl. The lower lip was nearly double its natural size, tumid, and rather everted. The epithelium was whitish and opaque, and constantly separated in distinct scales. There was also a superficial whitish ulceration on parts of the surface of the lip. The girl had enlarged and slightly ulcerated tonsils.

The affection of the tongue was observed in a middle-aged female. The tongue was enlarged, fissured in various directions, generally unnaturally florid, with here and there yellowish white ulcerations in hollows or in fissures.* The inner surface of the lower lip was similarly altered. The gums were tumid, soft, partially ulcerated.

These are the chief circumstances connected with the history of the ulcerated condyloma. I have endeavoured to portray them with conciseness and fidelity. There are several omissions of which I am aware—probably there are many of which I am not. The desire of avoiding prolixity may be offered as a partial excuse.

The treatment of the complaint is naturally divided into that of the ulcerated condyloma, and that of the secondary symptoms. A course of mercury is generally necessary for the former, almost always required for the latter. Prior to commencing it for either, the general health must be properly attended to. If the tongue is foul, or the bowels out of order, aperients must be given, indeed these may always be prescribed with advantage at the onset. If pyrexia is present salines should be exhibited. As soon as the secretions are

* There is no good account of the diseases of the tongue; yet the subject is one of great practical interest. I possess the notes of some curious cases. Probably Mr. Brodie could communicate much valuable information on the subject.

improved, and the general functions brought into good order, a mild course of mercury may be commenced. The blue-pill or the mercurial ointment are the preferable forms; five grains of the former twice daily, or inunction of half a drachm to a drachm of the latter, are the usual dose. Mercury should never be continued for any length of time without the combination of tonics. Of these sarsaparilla and good diet are the best.

The local applications and management are simple. If the parts are inflamed, tepid bathing and bread poultices may be employed. When this state is subdued, the black wash is the best application to sores; and, when these are healed, the oxymuriate lotion is useful.

To enter into further details would be tedious. Much of what was said with respect to the treatment of the simple condyloma is applicable to this. It would be out of place to dwell on the principles which should guide us in regulating a mercurial course. To these I shall advert very fully hereafter.

It remains for me to describe the third variety of condyloma. In this, the condylomatous deposites are not separate and distinct, but a mass of morbid structure is presented. The growth is chiefly seated in the cutis.

Its usual situations are the labia in females, and the perinæum, or vicinity of the anus in both sexes.

The growth is irregular—of variable size, but frequently extensive—fissured on the surface—sometimes, on the labia, possessed of little vascularity, and almost warty—generally, especially in the perinæum and round the anus, vascular and ulcerated, or secreting on the surface. The discharge, in the latter instances, is peculiarly offensive, and the part appears always in a slop. The varieties in form, which are chiefly displayed in the perinæum and around the anus, have given rise, amongst the older surgeons, to some curious terms. Thus, the names of *fici*, *crystæ*, *&c.* have been employed, from supposed resemblances borne to those objects by the condylomatous productions. This kind of condyloma is occasionally seen between the nates of infants, especially of such as are born of parents infected with venereal symptoms.

This form of condyloma may or may not be a venereal symptom. In some cases filth appears to give rise to it; in others it is clearly the consequence of discharge. It is occasionally followed by some of the secondary symptoms which have been described. In a female, who suffered to a great extent from this form of condyloma, a similar growth was developed in the axilla. The patient was cured of both. I have heard of other instances of this description of condyloma in the axilla; the above was the only one which I have seen.

After what has been said upon the other forms of condyloma, it would be repetition to dwell on the treatment of this. The same principles must regulate the management of all. As this form is frequently not followed by secondary symptoms, it is better to try simple remedies first. If the condyloma does not yield, or if secondary symptoms supervene, the propriety of a mercurial course will be more evident. Escharotics are more adapted to this than to the other varieties of condyloma. The arsenical lotion is very powerful and rather dangerous. If the oxymuriate lotion fails, perhaps the old and familiar combination of the powdered savine with the ærugo æris, will prove as good an application as any. Excision is sometimes adapted to the whole, more commonly to part of the condylomatous production.

A candid review of the preceding observations will, I hope, convince the reflecting surgeon, that the subject of condyloma is deserving of more attention than it seems to have hitherto received. The prevalence, and occasional severity of the affection, its varieties, its secondary symptoms, render it an object of much practical interest. Whilst it usually yields without much difficulty to a well-adapted system of treatment, it obstinately resists the random shots and the desultory attacks of inexperienced or ill-informed practitioners.

It will probably have occurred to the minds of some of the readers of this paper, that a striking singularity attending condyloma is its origin in gonorrhœal discharge, and its power of giving rise to secondary symptoms. We not unfrequently hear surgeons talk of gonorrhœal sores and gonorrhœal secondary symptoms. So far as my observation has extended, their ideas have been as vague as their language is indefinite. Unless condylomatous ulcerations be considered gonorrhœal, I have seen none, nor have I ever witnessed a fair example of secondary symptoms following uncomplicated gonorrhœa. Making no pretence to theoretic speculations, I merely offer the results of observation. If that observation be correct, condyloma would appear to form some intermediate state between gonorrhœa and syphilis.

The nature of the subject and the length of these remarks, preclude the necessity for the introduction of particular cases. I shall therefore proceed to offer a few observations on the subject of venereal warts.

### Venereal Warts.

They are frequent in both sexes. In the female, they occur on the outside and inside of the labia—at the orifice of the vagina—in the perinæum, and on the neighbouring portions of the thighs—in the groins, and round the anus. In the male, they grow from the inner prepuce and the glans.

The localities where they are found, and the circumstances under which they originate, prove that heat and moisture, and the lodgement of irritating secretions, natural or morbid, are the elements that favour their production. Some females exhibit a much greater disposition to the generation of warts than others. Those whose skin is coarse, and whose perspiration is more than usually offensive, appear to be more prone to these formations than individuals of a different conformation. Males whose prepuce is naturally long, are well known to be more subject to them than those whose glans is commonly uncovered.

Any thing which excites increased vascular action in the prepuce, on the glans, or at the outskirts of the vagina, appears to be capable of giving rise to warts. Thus, a patient who has recently had a sore upon the inner prepuce, very frequently discovers that warts have sprung up in the cicatrix. The same disposition is observed in the female. The majority of common prostitutes have warty growths at the orifice of the vagina.

It is probably for the same reason, that persons affected with chronic inflammation of the inner prepuce, a complaint on which I shall presently make some remarks, are also disposed to suffer from warts.

It is evident that warts are frequently independent of a venereal origin. But still more frequently they arise from gonorrhœal discharge.

Venereal warts display several varieties, but perhaps the leading division is into those which are highly vascular, and those which are comparatively little so.

The vascular wart attains a larger growth than the non-vascular variety. It usually springs, in the male, from the angle of the inner prepuce, and the glans. In the female, it is chiefly seen at the orifice of the vagina, the inferior commissure, the perinæum, and the margin of the anus.

It is soft, of a pinkish-red colour, lobulated, and composed of numerous productions, usually attached by a pedicle smaller than the superjacent growth, but sometimes arising by a broad base. When wounded, it bleeds freely. It is highly sensitive. It displays a remarkable facility of reproduction.

This form of wart not unfrequently occasions very troublesome consequences. When confined within the prepuce, it attains a large size, and gives rise to phymosis. The lodgement of the secretions then accelerates its growth, and aggravates the mischief. Inflammation and ulceration of portions of the wart occur—a sanious discharge is partly locked up, and partly escapes, from the præputial orifice—the inner prepuce is irritated, excoriated, inflamed, and ulcerated also—the ulcerated surface of the inner prepuce sometimes contracts adhesions to portions of the wart—sometimes the ulceration extends through both layers of the prepuce, and the wart or the glans protrudes through the opening, and sometimes a partial combination of all these circumstances is observed. This is not an ideal picture, for some of the most ill-favoured specimens of venereal diseases of the penis which I have witnessed, have been such cases as I have just described. On dividing the prepuce, or removing it, the glans resembles a cauliflower, in all but its purity of tint.

In the female, the vascular wart on the perinæum, or around the anus, is rather disposed in layers vertical to the surface than in rounded masses. This is the consequence of the lateral compression exerted by the nates and the thighs.

The non-vascular wart is not actually devoid of vascular organization, but the latter is much lower in degree in this than in the variety I have just described. It usually springs up in insulated growths. They are small, firm, numerous, and feel like granules. They seldom attain a much larger size than peas, but sometimes they run together, and form small, rough, brownish masses, with a pedicle much narrower than that of the vascular wart. This species is much less sensitive than the other. Patients may be seen to rub and pick off these warts without much apparent uneasiness.

The treatment of warts would appear to have exhausted the stock of escharotics in the materia medica. Astringents, acids, caustics, ligature, the knife, have been freely recommended and liberally used. I cannot pretend to add to the list of applications, but shall merely attempt to promote discrimination in their management.

There can be no question that frequent ablution will greatly tend to prevent the formation of venereal warts. In females with acute or chronic gonorrhœa, this is absolutely necessary. In males, whose prepuce is naturally long, and whose secretions from the glands, behind the corona, are apt to become irritating, the prepuce should be regularly retracted night and morning, and the glans and inner prepuce should be washed with a lotion of the acetate of lead. Lint dipped in this should be then applied, and retained, so as to separate the prepuce from the glans. By these means, warts will be generally prevented from appearing, or, if slight, will be removed.

But when warts have attained duration or magnitude, their cure is frequently a matter of difficulty. The following hints may perhaps be serviceable, in assisting the practitioner in his choice of remedies.

The vascular wart is more influenced by astringents than the other less organized varieties. Unless it is extensive, it frequently yields to the application of the undiluted liquor plumbi, which should be employed twice daily. Lint dipped in the same, or in a more dilute solution, should be constantly applied. If this is unsuccessful, I believe that a strong acid is the best application; it is certainly severe. If the vascular wart, however, is extensive, and the patient will submit to the treatment, the best and most expeditious method, is to conjoin the employment of the strong liquor plumbi with excision. The warts on the exterior of the mass should be excised with a pair of curved scissors, and the cut surface left should be touched with a piece of lunar caustic. By the combination of these means, a large mass of wart is very speedily got rid of.

The best mode of treating the non-vascular wart is by excision. Whatever substitute is tried, the surgeon most commonly resorts to this at last. The ligature is quite as painful, or more so, and certainly much less efficient. Whether excision or the ligature be adopted, the surface that is exposed should be touched with caustic. With this precaution, the wart very often re-appears—without it, we may generally count on its doing so.

When discharges co-exist with warts, proper means for arresting them should always be employed. Improvement of the secretions is desirable also—and for this, aperients, with the warm bath, and regulated diet are of service.

When warts of the glans, or of the inner prepuce at its angle, have occasioned phymosis, or those other consequences to which I have fully alluded, it becomes indispensable to divide the prepuce, or even to remove it. Indeed, if a patient with a prepuce naturally long, evinces a disposition to warts, which is not effectually prevented by cleanliness and the constant application of lint, I should strongly recommend the operation of division of the prepuce, or even that for the removal of some portion of it. I have performed both several times on this account, and the warts have subsequently ceased to appear. When adhesions have occurred between the inner prepuce and the warts, and ulcerated openings have formed in various parts of the prepuce, removal of a portion, at the least, of the latter becomes indispensably necessary. The mode of operation in the individual case must depend on the adroitness and judgment of the surgeon. It is impossible to lay down precise directions. In one case, in which the glans protruded at a lateral opening, an inch or an inch and a half from the free end of the prepuce, I introduced a director into the aperture, and passed it across till its point pushed out the prepuce on the opposite side. A sharp pointed bistoury was then run along the groove of the director, and the operation of removing the prepuce was completed by cutting off, at one stroke of the bistoury, the lower flap. Circumcision was thus very speedily effected without division of the prepuce from before backwards.

Perhaps, while on this subject, a few words may be permitted on the operation of division of the prepuce for phymosis, natural or morbid. It has been proposed by M. Cloquet to divide the prepuce at the side of the frænum, in order to obviate deformity. This method answers well enough in slight cases, where the quantity of prepuce to be divided is small. But in those in which the prepuce is long, or the constriction tight, this mode of operation is generally ineffectual. The reason is obvious to those who consider the anatomical relations of the part. The antero-posterior diameter of the glans is much smaller at the frænum, than on its dorsal aspect. The prepuce is consequently sooner reflected, and less is divided in the operation. If the portion behind the frænal reflexion is narrow, so much of the constriction remains of course.

When the operation is resorted to for warts, or for chronic inflammation of the inner prepuce, the plan of Cloquet is quite inadmissible. For the object in such cases is to expose the inner prepuce on the dorsum, and the method in question is incapable of doing so. The operation in short is seldom a good one, although it has been very highly recommended. The prepuce in cases of wart or of chronic inflammation should be divided at its middle on the dorsum.

I have probably said sufficient on the subject of warts. I shall conclude this paper with a few observations on an affection of the inner prepuce which frequently occurs in those disposed to warts, which, however, is more often independent of them, which deserves consideration because it is common and because it is productive of much inconvenience, and which I believe has never been described.

### Chronic Inflammation of the Inner Prepuce.

This occurs in persons whose prepuce naturally covers the glans. In some this almost amounts to phymosis, the prepuce scarcely admitting of retraction. In the majority of instances the prepuce either covers the glans very loosely, or only in a partial degree.

The inner prepuce in this affection is extremely red. The cutis is apparently thickened, or, rather, there is thickening in the cellular tissue external to the cutis. This thickening is made manifest by pinching the inner prepuce between the finger and the thumb.

There is a great disposition to excoriation, in fact, the patient is seldom free from superficial ulcerations of some description. When these are healed by appropriate treatment, the slightest indiscretion or neglect occasions their return. Venereal intercourse is frequently attended with severe pain; it almost always occasions abrasions, and frequently sores, which assume the elevated character, and commonly require mercury for their cure. Patients complain bitterly of the manner in which they suffer from connexion. One gentleman was separated from his wife for three years, prior to his placing himself under my care. He could seldom or never be connected with her, without the supervention of abrasions or of sores.

Not unfrequently warts arise from the inner prepuce in this state of chronic inflammation.

Irritation and enlargement of the inguinal absorbent glands appear as an occasional attendant upon it.

I presume that some natural irritability of the skin is the principal cause of this affection. Perhaps inattention to cleanliness may in some cases tend to its production. I have heard patients attribute its origin to gonorrhœa. This is not an improbable conjecture, for in that complaint it is not unfrequent for a limited portion of the inner prepuce to become red, irritable, and even thickened. With care and attention this state may be removed, but, it is reasonable to suppose, that without attention it may end in the condition of chronic inflammation.

To recapitulate. The chief characters of this affection are redness and thickening of the inner prepuce near the angle—tenderness of the part when touched, and, often, pain in it during coition—an extreme tendency to excoriations and to sores.

The treatment which readily occurs to the mind of the practical surgeon is ablution—the retention of lint between the foreskin and the glans—abstinence from all probable causes of irritation—and attention to the state of the secretions. But likely as it might seem, à priori, to succeed, this treatment is not in general successful. The part may become less irritable, the excoriations may be healed, but the slightest inattention or indiscretion is apt to render the complaint as bad as ever.

The only means with which I am acquainted, of effecting a cure of the affection, is division of the prepuce, or, which is even better, circumcision. I have performed both operations successfully on several occasions. If division is selected, it should be made upon the dorsum of the prepuce through the centre of the part that is the seat of the disease. If circumcision is preferred, and it seems to me the best, the surgeon should take care that he does not leave too much of the inner prepuce. The glans in either case should be perfectly denuded, and the inner prepuce at the angle should be bare.

For some time after the cicatrization of the wound, lint dipped in the solution of the acetate of lead should be worn around the penis behind the glans. The patient must be chary of venereal indulgences.

I must now bring this paper to a close. It has extended to some length, but I trust it will not seem to be composed of mere verbiage, or swollen by useless repetitions. I think it must appear, what it really is, the expression of what I have seen, rather than of what I may have read. Much as has been said on venereal diseases, there is room for the publication of the results of careful observation. Many who have written, have betrayed an imperfect acquaintance with the malady of which they treat—others have evinced a disposition to theorize, and exhibited the liveliness of their imagination, in lieu of the patience of their study, and the soundness of their judgment.

### LONDON HOSPITAL.

### DISLOCATION OF THE HEAD OF THE FEMUR INTO THE FORAMEN OVALE.*

Two cases of this description are related in the Lancet. As the mode of reduction which ultimately proved suc-

* Lancet, April 5th, 1834.

cessful was not the one which is most familiar, and most commonly applied, although it is similar in principle, it deserves to be recorded here.

*Case.* A man, æt. 35, was admitted into the hospital under the care of Mr. Andrews, having been thrown from a cart a short time previously;—he was not aware of the manner in which he fell.

One leg was widely separated from the other, and any attempt to bring them together caused considerable pain. The foot was in a natural position, without inclining to either side, and resting upon the heel. There was decided flattening of the hip, and increase in the length of the limb to nearly two inches. "On being removed to the operating theatre, attempts at reduction were made for more than an hour and a half, without success; when the operators were obliged to desist, in consequence of one of the hooks of the pulleys becoming straightened. Great difficulty was experienced in fixing the pelvis; it was therefore determined to defer all further attempts at reduction until two o'clock on the following day, when the patient was again brought into the theatre, and placed on the table. The pelvis was secured by round towels; and attempts at reduction were repeated, and persisted in for a considerable time, without success. At length the head of the bone was found to be removed from the position it had taken up, when the other hook broke, and the head of the bone returned to the place from which it had just been dislodged. The reduction was ultimately effected by placing the patient in a sitting posture, with a post between the thighs, when extension was kept up for a considerable time. The rope was then cut, and the limb at the same time being suddenly forced across its fellow, the head of the bone slipped into the acetabulum. The patient was in the theatre upwards of one hour and three quarters, the whole of which time was occupied in attempting to effect the reduction of the dislocation."

In the second case, the mode in which the accident occurred was highly characteristic. The patient was stooping, with the thighs widely separated, tying a rope round a sack of malt, which was to be lifted into the upper floor of a warehouse, when another sack was already raised to the upper part of the warehouse slipped from its rope, and fell across the lower part of the man's back.

The symptoms of the dislocation were obvious enough. Reduction was readily effected. The patient was placed in a sitting position with a post between the thighs. A round towel was then passed around the thigh, to which the pulleys were in the act of being affixed, when, on drawing the towel tight, the head of the bone suddenly slipped into its socket.

---

### London Dispensary.

### Ascites cured after Paracentesis.

In connexion with the case related by our friend Dr. Dickson, the following one, published by Mr. Robinson, in the London Medical Gazette, will prove interesting to our readers.

"Mary M'Key, æt. 26, transferred from the physician to me, August 27th, 1833. Appears of good constitution; the mother of three children. Labours short and natural. Was put to bed of her last child, 29th June. After delivery her abdomen was as large as before; her feet and ankles were rather swelled before delivery, and afterwards remained so. About the third day after the birth of the child she got up, and the abdomen increased in size. She was churched at the three weeks end, after which the abdomen still further increased in size, and she had some dyspnœa and cough. Elaterium and supertartrate of potash and gamboge were given; and on the 22d instant she rubbed in the strong mercurial ointment. Her mouth is slightly sore; the abdomen though considerably swelled, (forty-eight inches in circumference), is not very tense; there is a decided fluctuation on percussion. She has still a slight cough, but her breathing is free; respiratory murmur audible throughout the chest; slight rhoncus crepitans posteriorly and inferiorly, particularly on the right side. No fever; sleeps well. Pulse small, quick, and feeble; tongue clean and moist; bowels regular; urine copious; skin dry; faints at times on the least motion. Repetatur ung. nocte maneque; Ol. Ricini, ℥ss.; p. v. 2; Pulv. Digitalis, gr. 1-6th; Quin. Sulph. g. i. 3 die. The abdomen to be bandaged.

Sept. 3d. General health exceedingly good. Finds great support from the bandage; feels stronger; mouth sore; abdomen has gradually diminished, and is now reduced to 9½ inches; legs rather less swollen; milk deficient from the first; child much purged and greatly emaciated. Rep. ung. o. n. et Pulv. and bandage. A wet nurse.

7th. Child wonderfully plumped out since the change, and no longer purged. Mother steadily improving: legs have not swelled since last report, though she has sat up for an hour or two at a time: abdomen still decreasing, measuring four inches and a half less than on the 3d instant: a troublesome cough the last few days, relieved by linctus; mouth more sore. Some ptyalism. Repetantur omnia.

16th. Up, and goes out; ointment omitted; mouth still sore; abdomen two inches less; faint at times. Omitt. medicamenta.

Oct. 1st. Swelling of the abdomen rather increased, it is feared, but not proved by measurement; fluctuation more distinct; is going into the country. Repetatur unguentum nocte maneque.

Nov. 12th. Returned from country a few days; abdomen much more swelled and tense, 44 inches in circumference; umbilicus has started; the fluid passes into it, forming a fluctuating tumor, easily emptied; no œdema of legs; much dyspnœa; slight rhoncus crepitans throughout posterior part of both lungs; general health good; bowels opened yesterday by oil; paracentesis abdominis; ten quarts of green, clear fluid removed; no flakes of coagulable lymph; operation well borne; no visceral disease detected, though there seemed slight enlargement and hardening in the right lobe of the liver.

14th. Sitting up; easy; slept well; breathes much freer; cough trifling; mouth sore; several motions, with some griping.

Pil. hydr. gr. iij. Pil. sap. c. Opio, gr. ij. o. n. Inf. gent. co. ℥iss. Potass. acet. ℈ ter die.

Jan. 7th, 1833. Abdomen within an inch as large as when she was tapped; has not menstruated since her confinement; breathing unoppressed; legs do not swell; general health remarkably good.

Jan. 13th, 1834. She called at the Dispensary to day to inform me that she went into the country soon after last report; that the abdomen gradually decreased, and that she believed the fluid had entirely disappeared for the last two months. On a careful examination, there is not the least trace of fluid in the abdomen, nor any visceral enlargement; the abdominal parietes are flaccid; she finds great support from the bandage; goes about her work well; looks and feels in perfect health.

*Remarks.* There are a few points of interest in this case, to which I will briefly allude. 1st. I would ask to what was the dropsy attributable?—to pressure upon the large veins during utero-gestation; to disease of the liver; or to increased action in the vessels of the peritoneum?

That it is not entirely owing to pressure during pregnancy, is, I think, to be inferred from its return after paracentesis abdominis subsequent to delivery.

That it was not caused by hepatic disease I am also inclined to infer, from the patient's perfect restoration to health; from the few symptoms of hepatic derangement under which she laboured; and from enlargement or induration of the liver being suspected rather than proved.

I am therefore of opinion, that the dropsy was in this case owing to increased action in the vessels of the peritoneum, to which I am more especially led by the rapid return of the fluid after its removal—by its spontaneous disappearance subsequently—and by the circumstances which, in my mind, militate against the dropsy being produced by the before-mentioned causes.

I do not think the increased action amounted to actual inflammation, because there were no symptoms of peritonitis, and the fluid when withdrawn was quite transparent, and free from flakes of coagulable lymph—a circumstance by which I have frequently distinguished ascites with inflammation, from ascites the result of chronic visceral enlargement, or induration, a distinction sometimes difficult, but always important.

The good effects of mercury in diminishing dropsical effusions were conspicuous in this case, which is further remarkable from the palliative means proving radical, a circumstance rare in the treatment of hydrocele, and still more unfrequent in that of ascites."

---

### Dumfries Infirmary.

### Case of Empyema, with Operation. By J. Grieve, M.D.

The following case will shew that exploration of the thorax, by auscultation and percussion, is well understood in Dumfries.

*Case.* "Adam Lightbody, æt. 20, admitted into the Dumfries and Galloway Royal Infirmary, on the 25th of August, 1833—labours under constant dyspnœa, increased by motion and exertion of the slightest kind; difficult decumbiture on the right side; sense of fulness and oppression in the chest and abdomen. On percussion, the sound is clear over the superior portion of the right side of chest, and also over its anterior and posterior aspect; but over every other part of the chest, and particularly the left side, it is remarkably dull. Respiratory murmur inaudible, except in the upper part of right side of chest, where it is distinctly puerile. Pulsation of heart weak, and heard only on the right side of sternum. There is a decided difference in the size and figure of the two sides of the chest; the left being much enlarged, and measuring two inches more than the right, and considerably distorted from the uniform dilatation of the ribs, and increased width of the intercostal spaces. Integuments of left side œdematous, presenting a clear shining appearance. Abdomen enlarged and tumid, with distinct fluctuation. Right hypochondriac and epigastric regions sound dull on percussion, and feel hard and unyielding, evidently from enlarged liver. Lower extremeties are œdematous. Urine small in quantity, and high coloured. Bowels slow. Respirations 42 in the minute. Pulse 116—small and weak. He is harassed with a short cough, attended with scanty expectoration; no fluctuation is perceptible on succussion. Was exposed to cold nine months ago, and since that time his ailments have been gradually on the increase.

℞. *Hyd. submur.* gr. iij. *Pulv. jalapæ comp.* ℈ij. *M. habeat statim.*

August 26th. Bowels moved freely from medicine; in other respects much as yesterday.

℞. *Hyd. submur.* gr. ss. *Pulv. scillæ, Pulv. digitalis,* āā. gr. j. *ter die sumend.*

Aug. 27th. Passed a restless night, his sleep being interrupted by a sense of suffocation. Pulse 112. Respirations 30.

Aug. 28th. Dyspnœa much increased; abdomen more tumid and tympanitic; urine scanty, and rendered with difficulty; pulse 110; respirations 36.

℞. *Enema terebinth. habeat statim.*

℞. *Acidi hydrocyan.* gtt. iv. *h. s. sumend.—Cont. pilula, ut antea.*

Aug. 29th. One stool after enema, with discharge of flatus, which gave him temporary relief. Slept rather better. Left side of the body more œdematous—dyspnœa more urgent.

*Rept. Enema et Haust. anod. ut heri.*

Aug. 30th. Feeling of suffocation intense. Pulse 118, and feeble—in every respect is worse.

The symptoms were now so urgent as to preclude any reasonable prospect of his being relieved, otherwise than by paracentesis, which, after consulting with our colleagues, was determined on, and performed accordingly, in the usual manner. Upwards of fourteen pints of purulent fluid, of a yellowish colour, and most offensive odour, were drawn off; and during the issue of which, the respiration gradually became more free and unembarrassed, and he expressed himself much relieved. The fear that collapse might occur, deterred us from drawing off any more at that time. A tent was introduced into the wound, and a compress and bandage applied around the chest: an anodyne draught was administered.

We had now to learn, what he had hitherto unaccountably concealed, that he had been unable to follow his trade (a stocking-maker) for the last four months, in consequence of frequent pains and stitches in both sides of chest, but particularly in the left, for which he was largely bled, and had two blisters applied with relief:—and that two months ago, he expectorated, during two successive days, several mouthsful of matter, similar in appearance to that drawn off by the operation;—that in rising, or stirring in bed, he heard very distinctly a splashing noise in his chest.

Six o'clock, p. m. Continues tolerably easy; but not so much so as shortly after the operation. Respirations 32. Pulse 116; very irregular, and sometimes scarcely perceptible at the wrist. Faint respiratory murmur is now heard over the third rib or left side.

℞. *Liq. Morphiæ*, gtt. xv. *statim sumend.*

Aug. 31st. Died at four o'clock this morning.

*Autopsy.* On removing the sternum, we were surprised to find the cavity of the left pleura to contain very nearly *eight pints* of pus, making, with the *fourteen pints* previously drawn off, *the extraordinary quantity of twenty-two English pints!* The pleura was covered with an inorganic layer, of a white, curd-like substance, which could be detached with the handle of the scalpel, the membrane underneath presenting a grayish appearance, and apparently a little thickened. The lung situated by the side of the spinal column, under the cartilage of the third rib, was compressed and flattened, and reduced to the size of the clenched hand. When cut into it was dense and fleshy, presenting the carnified appearance described by some authors. At its lower margin was found a large tubercular abscess: a smaller one was also found in its upper portion. The heart was thrust from its natural situation completely over to the right of the sternum, and was covered with a thin layer of fibrine, which, in the left side, adhered firmly to the pericardium. The cavities of the heart were filled with coagula of fibrine, which was so firm and tenacious as to be drawn out with difficulty. The right auricle was nearly four times the size of the left. A considerable quantity of serum was effused into the right side of the chest—the inferior portion of the right lung had a coating of false membrane adhering at several points to the pleura costalis: the upper portion of the lung was infiltrated with bloody serum, and in several parts much indurated. In the left side, the diaphragm, in consequence of the incumbent fluid, was thrust down as far as the crista ilei, displacing the spleen and left kidney—the latter organ being placed by the sides of the inferior lumbar vertebræ! Both the kidneys were enlarged. Serum to the amount of three pints was effused into the abdomen. The liver was much enlarged; gall-bladder nearly empty; intestines naturally vascular.

*Remarks.* Considering the debilitated state of the patient—the extent of the effusion, and its long standing—*paracentesis* was perhaps a measure of questionable expediency; but the failure of all other means, and the rapidly increasing dyspnœa, rendered it our last and only resource. Had we possessed a more perfect knowledge of the history of this young man's case, and been aware of the tuberculated state of the lung, and of its complication with pneumo-thorax, as evidenced by his expectorating, some months before his admission, a quantity of matter, followed by relief of all the symptoms; and his being sensible of a splashing noise on any exertion or change of posture — we would have hesitated to sanction the operation under such inauspicious circumstances. There can be little doubt that, in this instance, in place of retarding, it rather accelerated the fatal event."*

Whether the operation accelerated the fatal event or not, it was perfectly justifiable. Were we in the same circumstances as the unfortunate patient, we should have implored the operation, even for the momentary relief it afforded. Life was not worth having under such sufferings—and the acceleration of death was a blessing. By the way, we do not see quite clearly that pneumothorax existed in the above case. Dr. G. does not state that air was found in the left pleural cavity, and neither the expectoration of purulent matter, nor the splashing noise which the patient heard, afforded positive proofs of pneumothorax.

---

### Guy's Hospital.

### Fractures of the Ribs and Sternum.

Mr. Bransby Cooper, in the volume of Surgical Essays which he has lately published, devotes one hundred and twenty pages to the consideration of various fractures. It might seem to require some boldness to embark on a venture of this sort, so soon after the publication of a work like that of our author's illustrious uncle. But any surprise must be diminished by the reflection that the pages of Mr. Cooper are chiefly occupied with clinical details and facts. The generalizations are neither extensive nor numerous. We have selected fractures of the sternum and ribs for notice upon this occasion.

Mr. Cooper observes that, in cases of fracture of the bony parietes of the thorax, when the serous cavity is opened, there is great risk of pleuritis. When the viscera are wounded the source of danger is threefold; first, from the effusion of air producing emphysema; secondly, from the great tendency to the effusion of blood; and thirdly, from the consequent disturbance to the important function of respiration. But Mr. Cooper has omitted to notice two other serious consequences which may arise from wounds of the lungs—empyema and pneumo-thorax. We have witnessed instances of both.

We perceive no remarks on the subject of simple and compound fracture of the ribs which need detain us. When the pleura is wounded in cases of fracture of the rib, the lung is generally implicated also. When this has occurred the treatment will depend on the quantity of blood which flows, and on the amount of difficulty of respiration.

"If the flow of blood be so great, and there be any difficulty in its escape from the chest, so as to lead to the dread of collapse of the lung from its pressure, it is then, not only wrong to close the external wound, but it should be enlarged, so as to admit of the free exit of the blood; which may be further assisted by placing the patient in the best position to facilitate its flow from the chest. Besides this, a considerable quantity of blood must be drawn from the arm, by a large opening, even to syncope, as the only means to be relied upon to check the continuation of bleeding from a wounded lung. Perfect rest, cool temperature, acidulated drinks, and every means on the one hand of diminishing the action of the heart, and on the other of maintaining the secretions, are to be employed; and as soon as there is the slightest appearance of reaction, indicated by pain, difficulty of breathing, and perhaps by some return of bleeding from the wound, the lancet must again be called in aid, as the only hope, dangerous as its use may appear, to save your patient, and thus allowing time for the healing of the wounded lung."

Mr. Cooper seems to deprecate much searching for extraneous substances in the chest, under any circumstances. If they are so near the surface as to admit of ready abstraction they should be withdrawn.

Mr. Cooper observes, with respect to emphysema, that the treatment consists in making free incisions through the skin to allow the air to escape; and in applying bandages through the whole extent of the distended parts, as well as over the ribs, to prevent perfect respiration. If compresses are employed,

* Liverpool Medical Journal, No. I.

they should not be applied opposite to the precise spot where the wound in the lung has been inflicted, for fear of forcing a fractured rib still deeper into the lung.

We think that our younger readers should receive these opinions of Mr. Cooper with some allowance. They should be told that emphysema, after injured lung, is not always frightful, nor always serious—that scarifications are by no means required in a number of cases—and that compresses are sometimes dangerous. We have seen four or five instances of emphysema after rupture of the lung, which did extremely well without any special treatment. We also saw a case a year or two ago in which emphysema succeeded a fracture of several ribs. It was attempted to apply a bandage on the chest, when the patient became affected with dyspnœa; his cheeks were bloated and purple; and he quickly died. We think that these remarks are necessary, in connexion with the description and recommendations of Mr. Cooper.

We shall now select some cases, in order to display the commencement and the end of all medical reasoning.

CASE 1.—*Fracture of the Clavicle and Ribs—Emphysema—Death.*

Susanna Webster, æt. 76, of delicate appearance, was admitted into Guy's Hospital Dec. 24th, 1832, a carriage having passed over her chest.

On examination, there was found a very comminuted fracture of the left clavicle, and fracture of several of the ribs on the same side, indicating laceration of the lung, by the appearance of an extensive emphysematous swelling. By placing the ear upon the left side of the chest, blood and air could be distinctly heard rushing from the lung. There was also a severe wound and contusion of the scalp, but no fracture of the bones of the skull could be detected. The respiration was very laborious; the pulse quick, small, and feeble; the extremities and surface of the body cold; the pupils perfectly sensible. A flannel was gently bound over the ribs and clavicle, and the wound in the head dressed; she was ordered the julep. ammon. every four hours, and bottles of hot water to the feet. Her state of collapse was so great, that no attempts were made to relieve the emphysema by incisions through the skin.

At 10, p.m. the pulse continued very quick and small—respiration irregular, frequent, and laborious—emphysema extending—the countenance anxious. Five drops of laudanum had been added, at half-past six, p.m. to each dose of the ammonia draught. Next morning it was necessary to draw off the urine by the catheter. At noon of this day (25th), respiration seemed to have ceased in the lower part of the left lung, and at 8, p.m. it could only be distinguished in its superior part. She sank progressively, and expired at 6, a.m. of the 26th.

*Dissection.* Considerable ecchymosis was found over the centre of the sternum, and on the ribs on the left side. The sternum was fractured at the upper part of its middle bone; the clavicle was also fractured in its centre, and much comminuted. The nine upper ribs were broken at their bodies; the fifth piercing the pleura, and lacerating the lung in two or three places; the sixth and seventh ribs were broken at their necks, and had pierced the pleura, which was found excessively inflamed, and within its cavity six ounces of blood which had been extravasated from the ruptured lung; the abdominal viscera were perfectly sound.

Mr. Cooper remarks, what must be evident to practical surgeons, that active measures were quite out of the question in a case like this. The age of the patient and the symptoms equally forbad them.

The next case is one of a less serious character. The patient, a coal-porter, aged 56, fell over a coal-box while in a state of intoxication. He was admitted, complaining of great difficulty and pain in respiration, which was short and quick; his face was livid; his pulse quick and feeble, and his extremities were cold. On examination of his chest, three ribs were found fractured on the left side, between the eighth and eleventh. He was immediately put to bed; warm bottles were applied to his feet, and warm tea given him to drink. In about two hours reaction came on, a flannel bandage was then tightly applied round the chest, and as he had entirely recovered from his previous state of depression, complaining also of pain in the seat of fracture, he was bled to the amount of thirty ounces. Purgative medicine, the antiphlogistic regimen, and a cough linctus, were sufficient to effect this patient's cure.

Mr. Cooper insists on the necessity of waiting for reaction before active means are employed. Nothing certainly can be more ridiculous than bleeding a man because he is knocked down or run over, without stopping to consider his probable injury, or the actual state in which he is. But we fear that too many are inclined to run into the opposite extreme, and to exhibit cordials and restoratives in order to induce reaction. They blow the fire they must soon be at considerable trouble to extinguish. It is impossible to lay down a rule so general as at once to comprise the various particulars and apply to each. We would say, avoid endeavouring to induce reaction in cases of severe injury, unless there is decided danger from the mere collapse. When this is the case the injury must in general be so grave, that the result is much the same whatever may be done, but certain we are, that we have seen much mischief in cases of injury of the chest and abdomen, from the over-anxious and officious endeavour to procure reaction. Quiet and moderate warmth are amply sufficient in the great majority of instances.

Mr. Cooper relates an interesting case of laceration of the lung.

*Case.* W. M., æt. 18, a sailor, was admitted into Guy's Hospital, Oct. 19th, 1831, having fallen from the mast-head of a vessel on a belaying pin attached to its side. The peg had entered the chest just above the clavicle, and, having penetrated upwards of seven inches, had suddenly broken off. Mr. Randall, of Rotherhithe, extracted the peg, which required the application of great force, and was followed by considerable hæmorrhage. He was then taken to the hospital.

On admission, he complained of great pain in his left shoulder, and an uneasy sensation in his abdomen. There was considerable tumefaction of the left side of the face and eye-lids, with ecchymosis. A large lacerated opening immediately above the clavicle, about four fingers in breadth, presented itself, through which the clavicle might be felt fractured into two or three portions, and the subclavian artery was perfectly laid bare, as it passed over the first rib. There was emphysema extending from the neck, down the side and back; the surface of the body and feet cold; the abdomen somewhat distended, and he had partial priapism. The pulse 140, small, and sharp; he was ordered to be kept perfectly quiet. The edges of the wound were brought together by straps of adhesive plaster, and a dossil of lint applied to absorb the oozing blood; he was ordered to take the julep ammon. with the hopes of producing reaction.

At 8, p. m., the pulse was 120, fuller. He complained of increased pain in the abdomen. He was bled to 18 ounces, and a quart of urine was drawn off by the catheter. After this he sank. Constant sickness came on, and at 5, p. m. of the 20th, he died.

*Dissection.* "The wound produced by the penetration of the peg, extended from immediately above the left clavicle into the axilla; the clavicle itself was fractured in two places, and the subclavian artery, or more anatomically speaking, the axillary, was found lacerated; a large opening was found passing from the axilla, into the chest between the third and fourth ribs, leading into the left lung; and a large piece of blue cloth was firmly fixed into the lung itself, forming a plug which probably prevented immediate death from hæmorrhage. This wound was found to extend to the diaphragm, which presented a very ecchymosed surface; the spleen was found lacerated on its inner and posterior surface. The stomach and intestines were filled with air."

During life pulsation had been felt in the radial artery at the left wrist,

It was found, upon close examination, that the subclavian divided into a radial and ulnar branch, immediately below the first rib. The ulnar branch only had been wounded.

We extract the following directions with respect to treatment laid down by Mr. Cooper.

"When fracture of the ribs is indicated by an acute pain in respiration, the treatment consists in passing a broad girth with a buckle and strap tightly around the chest, so as to keep the ribs stationary during respiration. The surgeon, however, should first examine whether the salient angle, produced by the fractured extremities of the ribs, be directed outwards, or inwards towards the lungs; if outwards, a compress of wetted linen should be applied over the part fractured, but if inwards, two compresses should be adjusted at a distance from the fractured part, and in such a situation as may tend to press the displaced portions outwards from the cavity. When the ribs are fractured on both sides of the chest and opposite to each other, a bandage ought never to be applied, as it would press the fractured ribs upon the lungs: in such cases, bleeding, and the strictest antiphlogistic regimen is to be trusted to."

Mr. Cooper relates a case of fracture of the sternum, or rather of separation of its first and second portions, which deserves commemoration.

*Case.* A man, aged 23, was admitted into Guy's Hospital with displacement of the upper end of the second piece of the sternum, which was driven by a blow behind the first piece. Immediately after the accident he suffered from dyspnœa, and experienced considerable pain on drawing a full inspiration. Upon examination, the extent of injury was quite obvious, but no means which could be employed could bring forward the depressed portion of the bone; the object therefore was, by constitutional means, to prevent inflammatory action, and by the mechanical adaptation of a bandage rolled around the chest, as in fractures of the ribs, to prevent the motion of the displaced bone; bleeding, purging, and the strictest antiphlogistic regimen were adhered to; and upon the application of the bandage, a compress was placed upon the lower part of the sternum, so as to have a tendency to press the displaced portion forwards. These means immediately produced the desired effect, removing all difficulty of breathing; and in a few days after, while in the act of coughing, the depressed portion was forced into its natural place, and the man, from that moment, was convalescent.

We once saw a case of fracture of the sternum occasioned in a curious manner—by the patient's pitching on his head. The upper portion was displaced, we think backwards. The only means of remedying the malposition was by keeping the head well back, when the sterno-mastoid muscles pulled up the displaced portion of sternum to its place. The remedy, however, was productive of more inconvenience than the injury, and the malposition was permanent.

"It sometimes happens," says Mr. Cooper, "that inflammation extends into the anterior mediastinum, affecting the absorbent glands, or perhaps, the remains of the thymus gland itself, which may go into a suppurative state; producing dyspnœa from pressure upon the lungs,—disease of the sternum from its tendency to be evacuated externally, —and presenting a pulsating tumour from its contiguity with the heart.

Such a case occurred in the practice of Sir Astley Cooper, in the person of a medical student, who believed himself, and had been led to believe by other medical men, that he was the subject of aneurism; under which conviction he had made up his mind to make a voyage to Bourdeaux, there as he believed to die. Before he left London, however, he thought he would consult Sir Astley Cooper, who told him at once the nature of his complaint, evacuated the matter, which had made its way through the sternum, and removed, simultaneously, both the appearance of aneurism and the fears of the patient."

There are other portions of this volume to which we shall refer upon future occasions. Practical facts and observations will always be valuable.

### Tralee Hospital.

### Pneumo-Thorax, from Perforation.

In the May number of our esteemed Dublin contemporary, Mr. Poole, assistant surgeon of the 32d Regiment, relates a case of the comparatively rare disease at the head of this article. The subject of it was a young soldier, of healthy appearance, admitted into the Tralee Hospital on the 27th of March, 1832. The chest was not well formed. He had had pneumonia two months previously, and was affected at the time of entry with violent cough, dyspnœa, viscid expectoration; but without any loss of resonance, or other sign of disorder in the chest, except some mucous rattle in the lower left lobe of lung. The disorder was considered bronchitic, and treated with antimonials, light diet, and counter-irritation. He improved much, and was convalescent. On the 27th April, however, the lower portion of right side gave a dull sound on percussion, and the respiratory murmur was heard indistinctly, but without any aggravation of symptoms, till the beginning of May, when the cough again became troublesome, and the aspect generally unhealthy. On the 12th May, loud bronchial breathing was heard in the mammary region of the left lung, with bronchophony, but no ronchus, the resonance continuing. On the 21st, a considerable change took place, the patient having been attacked with pneumonia of the lower part of the left lung, which yielded to bleeding and antimony, but not without leaving hepatization.

" After the signs of this latter lesion had entirely disappeared, a mucous râle persisted through the whole of the posterior portions of the lung, the anterior part giving a clear sound on percussion, until the 1st July, when the metallic tinkle was distinctly heard, and a diagnosis of pneumo-thorax made, although the respiratory murmur and a deep gurgling could be distinguished in the portions of the lung, over which percussion elicited the clearest sound. There never existed any signs of an excavation, but the patient coughed up, on the 4th and 5th of the month, a large quantity of a sero-albuminous fluid, proving that a communication existed between the lung and the sac of the pleura. From this time he sank rapidly, and expired on the 6th of the month.

*Autopsy twelve hours after death.*—Considerable marasmus; left side of the chest, at the inferior portion, much bulged out, highly sonorous, in fact, tympanitic; right side gave generally a dull sound on percussion; on cutting through the cartilages of the left ribs, a rush of inodorous gas took place from the pleural cavity. The lung was found condensed, and pressed up towards the spine and back part of the ribs, to which it was bound by strong adhesions. A small quantity of a yellow coloured serosity existed at the bottom of the cavity. The surface of the lung was covered with several loose layers of false membrane, of a lemon colour and tough consistence; they were readily detached from the lung; the lung was firm and corrugated, it was perforated by a considerable opening, which existed at the back part of the upper portion of the lower lobe, close to the adhesion with the costal pleura; the finger could be passed by this opening into the substance of the lung, and it was found that a small quantity of purulent matter escaped by it when the lung was pressed. The parenchyma of this lobe was thickly infiltrated with tubercular matter in a crude state; one or two points of suppuration were observed, but anteriorly there was no excavation. On the back part of the lobe, however, corresponding with the situation of the fistulous opening, existed a considerable irregular cavity, which had through this discharged its contents into the pleural sac. The upper lobe of this lung was quite sound, and did not contain a single tubercle, but all its bronchial tubes were much dilated, their lining membrane highly vascular, without any apparent hypertrophy. The sac of the right lung contained some serosity, but the lung itself, with the exception of its lower lobe, which was thickly studded with crude tubercles, was sound and crepitous."

### MEATH HOSPITAL.

### SURGICAL REPORT OF CASES TREATED DURING THE PAST YEAR. By W. H. PORTER, Esq. &c.

The Dublin surgeons, Mr. Crampton, Mr. Carmichael, Mr. Porter, and others, have done themselves much credit by the readiness they have evinced to communicate clinical facts to the profession. Yet we think we perceive some indisposition to exertion creeping over the energies of the Irish hospitallers.

Mr. Crampton's throat is dry, and the surgeon-general has not lately poured forth his clear and sonorous chirurgical strains. We shall quarrel with these gentlemen, if they now turn laggards. It must not be.

A criticism on a case of Mr. Carmichael's has been taken in ill part. We know not the exact phrenological explanation of a fact which we have noticed, but the fact itself is a good one notwithstanding—that, in our critical experience, we have never seen the man who thought that he was over-praised, nor him who did *not* think a criticism on himself ill-natured.

If there ever was a medical journal in which approbation has been liberally, handsomely, gratuitously given, it is this. None are more disposed than its conductors to take the will for the deed —none give the generous cheer more loudly nor more long. Yet the instant they attempt to criticise, be it ever so considerately, they are overwhelmed by complaints of unkindliness and cruelty. The writer and his friends should, however, reflect, that the reviewer's *must* be the ungentle craft, and that his ink grows colourless, if it is not mixed with gall. Unless his pen, like—

> Ithuriel's spear,
> Still shews the cloven foot—the lengthen'd ear, it is decried by the public for an useless stump, and its characters wear neither form nor impress. For ourselves, we can only say that it always gives us pain to utter even a just criticism, and we appeal to our readers for the general candour, manliness, and generosity of our remarks.

To return to Mr. Porter and the Meath Hospital. We cannot forbear from introducing his prefatory observations, displaying, as they do, the good sense of the writer, and offering some excellent hints to reporters and to authors.

"The general practice of all hospitals," says Mr. Porter, "must be nearly the same, and as the leading characters and treatment of the ordinary forms of disease are pretty accurately understood, very detailed reports are neither necessary nor instructive, unless when adduced for the purpose of establishing some important pathological fact, or introducing some improvement in practice. But in every establishment of this kind, particular cases will occasionally occur, not only novel in their nature, and therefore curious, but by reason of their infrequency, difficult and uncertain in their management. By the publication of such cases in an authentic form, the hospital surgeon may confer the greatest benefit on his profession, for he enables the practitioner, of extensive opportunities, who has met with similar cases, to compare the observation and experience of others with his own, and thereby approach the truth; whilst to the younger practitioner, he furnishes a guide and assistance in the difficulties of his profession, which, though far from perfect, may nevertheless be valuable. In this spirit and with this view, I have selected the following cases, each possessing its own peculiar interest, and on which I forbear to offer comment or observation, wishing to render the details as short as shall be consistent with clearness; and knowing that the case, which to the practitioner at the bedside will appear rare, or difficult, uncertain, or important, may to the reader in his closet, deprived of the numerous aids derived from personal inspection, seem to possess little more than ordinary interest, and unworthy of being obtruded on the profession."

It appears to us, that the object of hospital reports is not merely to publish extraordinary cases, or to chronicle remarkable improvements, though both are highly desirable and necessary. It must be remembered, that a large portion of the medical public is placed beyond the reach of those opportunities for maintaining a high standard of professional knowledge, which so amply abound in large cities. The surgeon, in thinly-peopled districts, or in our colonies, has no access to hospitals—no medical society—neither leisure nor opportunity, nor perhaps inclination, for laborious reading. His chief source of information is a medical journal, and on this he must depend for keeping him near the level of his brethren in the towns. He does not require to be told of extraordinary cases, which may never fall under his personal observation; but he looks for details of the current practice, for records of those facts which occur in masses, and constitute the sum of useful practical experience.

We think that hospital reports, if systematically given, would afford more valuable information to the profession, and diffuse more widely a knowledge of the true principles of reasoning, than any other kind of publication. The reader would acquire that taste for facts, and that disrelish of all not simply deducible from them, which tend, more than any other mental exercise, to give a bold and independent bearing to the intellect, to encourage acuteness of the observation, and to confirm the soundness of the judgment. The hospital reporter should endeavour to present a faithful picture of the varied character of hospital experience. He should not select the striking cases only, for they are the minority in nature; but, on different occasions, he should attempt to present the different orders of facts which are really met with in the hospital wards. If, influenced by the modesty of Mr. Porter, all reporters were to refrain from noticing the common cases, the public would be feeding on unnatural and stimulating viands, which must corrode their taste, instead of affording it wholesome nutriment. It is as if every host should offer only brandy to his thirsty guest, because he might procure pure water elsewhere.

In accordance with his plan, Mr. Porter relates four cases, severally possessed of some peculiarities. They are these:—1, Aneurism occasioned by the sequestrum, in a case of necrosis of the tibia—2, Functional derangement of the brain, the result of injury, cured by the operation of the trepan—3, Curious and interesting case of bronchotomy—4, Disease of the lymphatics of the left arm, demanding amputation at the shoulder-joint.

These cases we shall notice in regular succession.

Case 1.—*Aneurism in a Case of Necrosis—Death from Mortification and Hæmorrhage.*

A man, æt. 29, of drunken habits, admitted January 2d, 1833.

His face was blanched and anxious. On uncovering the limb, a small livid fistulous opening was seen on the outside of the lower third of the right thigh, slowly discharging a thin, serous blood, on pressing which the finger seemed to sink into a deep cavity; pulsation was quite distinct, and bruit de soufflet audible for some distance round it, as if from aneurism: the femur, at its lower third, could be felt enlarged, and the popliteal space filled up, but the pulsation of the artery below it was distinctly perceptible. There was intense pain in the knee, and throughout the tumor—extreme exhaustion—pulse 150, small and thrilling.

The disease seemed to have begun fourteen or fifteen years previously, with violent pain in the knee and lower part of the thigh, which rapidly swelled to a great size. The tumefaction diminished under the use of blisters. About a year afterwards, a small swelling appeared four or five inches above the knee, which was opened, and gave exit to some purulent matter; a sinus remained there ever afterwards. In August, 1832, an alarming hæmorrhage occurred from the fistulous opening, and this returned on the night preceding his admission into the hospital. The patient supposed that he had lost several quarts of blood, and he fainted from exhaustion seven or eight times.

From all these circumstances it was conceived that the case was one of popliteal aneurism complicated with diseased bone, the sac having probably burst into the cavity of an abscess in connexion with the bone.

A compress of lint was placed over the opening, and a bandage rolled from the foot, over and above it. Lemon juice was given ad libitum.

Some bleeding occurred during the night, and next day he was seized with severe vomiting; pulse 150, full and hard. On the 4th there was intense pain in the thigh. The face was bleached, with a yellowish tinge, and extreme anxiety — thirst urgent — pulse 142. Amputation was obstinately refused by the patient. The vomiting continued — the debility increased — and on the 6th there was great swelling of the thigh, with gangrene on its posterior surface, nearly as high as the buttock. A constant though feeble hæmorrhage trickled from the limb. At 9, p. m. he died.

*Dissection.* "On opening the popliteal space it was found filled with thick grumous clots, extending up as high as the lower third of the femur, in contact anteriorly with the bone, and with something that appeared to be part of the sac, but whether of an aneurismal sac, or the cyst of a former abscess, could not be determined. An opening existed in the popliteal artery, a little below the spot where it enters the space. The thigh-bone was found diseased in its lower half, being considerably enlarged, its surface rough, and a large portion of the posterior or popliteal aspect destroyed, so as to permit the introduction of the fingers into a large cavity within; the edges of the bone on each side of this opening were thick and very full of rough sharp points; in the upper part of the excavation the sharp point of a sequestrium was discovered, moveable, and accurately corresponding to the aperture of the artery, which it evidently seemed to have occasioned. The knee-joint filled with a yellowish serum, unlike ordinary synovia; its capsular ligament thickened. The cellular tissue of the entire thigh filled with a reddish serum."

There is slender evidence and but slight probability of the above having been an instance of true aneurism, that is, of the artery having been dilated into a sac, prior to a laceration or wound of the latter. It is more consistent with the circumstances to suppose that the wound was of the artery itself, and that the blood became diffused into the cavity of the abscess or surrounding cellular structure.

CASE 2. *Fracture of the Os Femoris, followed by Symptoms of Idiotcy and Paralysis—Recovery after the Operation of Trephining.*

Ed. Hughes, æt. 35, a healthy countryman, admitted June 26th, 1833.

On the 8th of the previous May, he was knocked down by a blow of a large stone nearly in the centre of the forehead, which produced a depressed fracture of the frontal bone. He was not rendered senseless by the blow, nor for sometime afterwards was there any perceptible consequence, as during the six subsequent weeks he was able to work, and had his intellects perfect and natural. His friends then observed him to become drowsy, listless, and incoherent: when undisturbed he was quite idiotic, but when roused he appeared to possess some memory. His manner of answering a question was very remarkable; he hesitated, seemed to recollect with difficulty, and answered as if in doubt. He tottered in his gait, and had a remarkable tremor in his left arm and hand: the tongue, when protruded, was drawn to the left, and there was also strabismus of the left eye, symptoms which appear to have been present at the time of his entrance into the hospital.

Mr. Porter, under these circumstances, cut down upon the bone, and found an irregular fracture of nearly an inch in length, one side of which was depressed to the depth of little more than three lines. The trephine was then applied, in order to permit of the elevation of the depressed bone, but the internal table was found to have been so extensively broken, that three crowns of the instrument were removed before all the fragments could be exposed and taken away; one large portion had penetrated the dura mater, and entered the substance of the brain, the removal of which was followed by considerable hemorrhage, appearing to come from some vessel of the brain itself, and which could only be controlled by the application of several compresses, and such a degree of pressure as evidently affected the functions of the organ.

During the remainder of the day the patient continued listless and half asleep. The pulse was slow, small, and weak. On the next day he was nearly insensible to external objects; the evacuations were passed involuntarily; the pulse was weak and labouring. On the removal of the compresses and dressings he became more lively, and his pulse more full. On the following day (June 30th) he appeared much more sensible. The pulse was 86, and strong. On the 1st July, the patient became sensible, for the first time, of his natural wants. The pulse was 110.

He progressively improved until the 16th, when he had several severe rigors and trembled violently. He was drowsy and stupid—the eyes were peculiarly wild—the pulse rapid and fluttering. He again became unconscious of his natural wants, and fell into nearly an idiotic condition. The tremors were remarkable, especially those of the left arm and leg. The bottom of the wound was covered with white granulations, resembling fungus, and the discharge was very profuse and rather fetid. The treatment for the previous symptoms had been strictly antiphlogistic. He was now ordered calomel and opium so as to affect the mouth.

In proportion as the mercurial action was developed, the symptoms passed away, and on the 28th, the patient was pronounced convalescent. After his recovery, he is said to have remembered most of the circumstances that occurred during his illness, which is rather extraordinary, considering that he is described to have been unconscious of his natural wants, to have lain in a kind of slumber, and to have been nearly idiotic.

In the November following, the patient was so well as to walk to Dublin, a distance of twenty-three miles.

This case seems to display the good effects of mercury in the secondary inflammation of the brain and of its membranes, which ensues after injuries of the organ, and operations on the cranium. We have drawn attention to this fact upon several occasions, and we cannot take a better opportunity of enforcing it, than by reference to the particulars of the case before us. It is more than probable that, but for the mercurial influence, the patient would have suffered from formation of pus, the consequence of inflammation within the cranium.

Case 3. *Bronchotomy for the removal of a Foreign Body, supposed to be in the Larynx.*

James M'Mahon, æt. 14, admitted Aug. 27.

In the previous June, whilst eating some beef-hash, a piece of bone or gristle seemed to have stopped in his throat and he was instantly seized with all the symptoms of suffocation, violent cough, &c. He remained in this state for some days, with great pain and difficulty of swallowing, and of turning the head to the left side. He was taken to a hospital, where a probang was passed down the œsophagus with slight temporary benefit. The symptoms soon returned with violence. The following are the symptoms observed on his admission into the Meath Hospital.

"Deglutition so difficult and painful as to make him refrain from drinking, although very thirsty; he cannot turn his head to the left side without great suffering; voice nearly lost; breathing loud and sibilous; the wheezing greatly increased during the spasmodic paroxysms, which are very frequent. The face is pale and livid, the lips purplish. He opens his mouth badly, but as far as the condition of the fauces can be ascertained, there are no traces of inflammation. On passing the finger into the fauces, the epiglottis can be felt of its natural size, and healthy. Pulse rapid; skin hot and dry."

Supposing that some foreign body was impacted in the larynx, Mr. Porter proposed the operation of tracheotomy, to which the mother refused her consent for two days, when it was granted.

The operation itself proved extremely difficult; and, although much thick mucus was expelled, the patient was little, if at all, relieved by it.

We need not pursue the detail of symptoms. It is enough to say that, whenever the wound became obstructed by mucus, the dyspnœa was frightful, and productive of immediate danger—that it was necessary to have a tube in constant readiness for introduction upon such occasions—that respiration was solely effected by the wound, and was arrested by its closure, though a large-sized bougie could be passed through the rima glottidis—that gradually the paroxysms of suffocation grew less frequent—that the patient had two attacks of bronchitis—that, after the lapse of some months, the passage of air through the larynx could be effected, and the patient could remain with the tube closed for half an hour, or for more—but that even very lately, when Mr. Porter saw him, he did not dare to sleep without the tube in the artificial opening.

As a foreign body was never discovered nor expelled, and as a probe could be passed through the rima glottidis, when the difficulty of breathing was at its utmost height, it seems reasonable to suppose that the latter symptom was chiefly due to spasm of the muscles of the glottis, kept up, perhaps, by inflammatory action in the larynx. This, however, is conjecture. The fact remains, and a curious one it is.

CASE 4.—*Disease of the Lymphatics of the Arm—Amputation at the Shoulder-joint.*

Michael Hughes, æt. 29, admitted Nov. 27th, 1833.

"The limb is semi-flexed and slightly swollen at the elbow; the hand bent downwards at the wrist; the knuckles bent also, and every one of these joints permanently rigid. There is an eruption on the hand, which is itchy and troublesome. The veins of the arm swollen and prominent, but not hard or knotted. The pulse at the wrist so weak as to be scarcely perceptible.

An irregular line of small tumours, resembling tubercles, extends along the arm from the axilla in which the largest is situated; they are of a stony hardness, and firmly attached to the adjacent structures; some of them are ulcerated, others surrounded by a dark blush of inflammation. The pain he experiences is dreadful, and deprives him entirely of rest. He entreats that something may be done for him, and will submit to any operation that holds out even a chance of relief. The history of this case is shortly this. The tumour in the axilla was the first formed, and appeared four years previously, after a day of very hard work; its growth was slow and irregular, sometimes increasing, sometimes diminishing, and never very painful. The gland next below it then swelled, and thus the disease continued to spread along the arm in the direction contrary to the course of absorption."

Several months previously, he had been in the hospital under the care of Dr. Graves, who prescribed iodine and other remedies without the slightest benefit. He quitted the establishment on hearing of the possibility of an operation.

On the present re-admission, Mr. Porter determined to perform amputation at the shoulder-joint, although the patient had a slight cough, and the expectoration was triflingly tinged with blood. It was found, during the operation, that the artery, vein, and nerves were encompassed by the tumor in the axilla. The patient left the hospital at the end of five weeks, but he continues to suffer from pectoral symptoms, and his ultimate recovery may justly be considered as highly improbable.

The dissection of the arm exhibited a number of the lymphatic glands indurated, others ulcerated, but all exhibiting proofs of their malignant nature. Several glands, however, were found healthy, and without any alteration of structure. One in the axilla, close to the large original tumor, was slightly increased in size, and softened in consistence. The artery, vein, and nerves had passed through the centre of the indurated gland, and the former vessel had been so compressed, that its calibre scarcely equalled that of a small crowquill. The axillary vein was also compressed, whilst the cephalic was increased in size. Having passed the tumor, the artery again seemed to expand into its natural size, but still the compressed state of the vessel was indicated before the operation, by the smallness and feebleness of the pulse. The distention of the superficial veins shewed, that the circulation through the deeper trunks was more or less impeded.

Mr. Porter supposes that the eruption on the hand was connected with the condition of the nerves. It is perhaps more likely to have depended on the state of the circulation and absorption. In three cases of subclavian aneurism, Mr. Porter has observed a similar eruption and crookening of the fingers, which were removed after the operation, when the pressure of the tumors was no longer exerted.

"I had intended (says Mr. Porter) to have added some other cases to this report, as having occurred within the past year: amongst them that of the man whose face was nearly eaten off by a pig, but independent of the length to which these details have been already drawn out, I find, that the interest of this case would not compensate for the disgust its perusal would occasion. It merely tended to shew, first, that a man deprived in this rough manner of his nose, both cheeks, both lips, and part of both ears, might recover notwithstanding; and secondly, it exemplified the uses of the lips to the functions of speech, mastication, and deglutition. It was remarkable, that soon after the accident, he could pronounce the labial letters tolerably well, a power which he lost as the wound progressed in healing, and of which he was entirely deprived before he left the hospital. His articulation of course was very indistinct. He lost more than half of the solids he attempted to chew, for want of the lips to keep them under the teeth, and a considerable portion of the fluids escaped in his attempts to swallow. His chief regret was the being deprived of the use of tobacco, which he was incapable of using in any form, on account of the loss of his nose and lips. All the saliva secreted by the parotid glands escaped, and several attempts were made to ascertain the quantity produced in a given time, but they were defeated by the irritability of the patient. Although deprived of so large a portion of this fluid, I did not find that the process of digestion was at all impaired."

We feel obliged to Mr. Porter for his report. We trust that he will persevere, and that many of his compeers will not draw back from the good work.

---

### Glasgow Royal Infirmary.

### Clinical Report on Erysipelas. By Dr. Macfarlane.

The succeeding cases are contained in the volume of clinical facts published by Dr. Macfarlane. To that volume we have referred upon several occasions, and to it we shall take convenient opportunities of referring again. Cases such as those of which it is made up, are not of a day, but of all time. They do not possess the ephemeral interest that attaches to novel speculations, or new-furbished theories, but may be studied when the false gloss of novelty is rubbed off, and when truth displays its simple and its sterling qualities. The great object of the Editors of this Review has long been, and will still be, to endeavour to direct the taste of its readers to inductive reasoning—to point out the intrinsic value of facts, and the meretricious vanity of hypothesis. They venture to hope that their labours have not been altogether unsuccessful; and they feel encouraged to persist in the attempt at working a substantial reform in practical medical literature. There are some who misconceive, and many who resent their conduct—who think, or who affect to think, that their disregard of theory is founded in critical perversity—their expressed contempt for speculation in indolent levity. These gentlemen may have their way. They are adapted for the olden days of doctrines and of systems, but they cannot comprehend the spirit of present science, in its genuine sense. Let them worship at the shrine of Hippocrates and Galen.

Erysipelas is a disease in which the absurdity of dogmatism is apparent. The nosologists scarcely know where to place it—the systematists cannot tell how to treat it. One informs the timid and wavering student that it is a purely inflammatory malady, and that bleeding, purging, antiphlogistic remedies of all kinds, are indispensably required for it. Another exclaims that this practice is murderous—that erysipelas is atonic, asthenic—that bark and brandy are necessary to enable the patient to fight against it. The modern surgeon might despairingly inquire, with the ancient sage—Where is truth?

When we avert our eyes from this dogmatic warfare, and turn to the contemplation of nature itself, we find no exclusive theory supported. Her facts are various—her objects are diversified. Erysipelas is not observed to display universally the same characteristics, any more than the constitutions and the habits of its subjects are found to be precisely of the same description. The pale countryman and the squalid mechanic of the crowded town's most crowded lane, are perceived to be both affected by the malady: and none but a Laputan sage,* or a medical Philosopher Square, could suppose that persons in such opposite circumstances, of such opposite degrees of constitutional power, could exhibit similar phenomena, or require similar treatment. Mr. Lawrence, a vigorous advocate for depletion, and a cordial opponent of stimulating treatment, under almost any circumstances, has adopted a singular argument on this occasion. As he cannot altogether deny the fact, that the constitutions of persons in the country and the town do exhibit some dissimilarity, he has asked triumphantly, where we are to draw the line? at what mile-stone the district of the countryman begins, and that of the Londoner ends? if the resident of Brompton should be bled or barked? This may be witty, and is fallacious. The ready answer to Mr. Lawrence's inquiry is this:—Go study Nature, and see where she has drawn the line—disregard the mile-stone, and feel the pulse—ascertain the previous condition of the patient—observe the actual characters of the attack—if his face is flushed, his skin hot, his heart beating vigorously, his tongue white, and especially if symptoms of inflammation of some organ are displayed, then resort to antiphlogistic treatment; but if, upon the other hand, you find him emaciated, pallid—his pulse weak—his tongue trembling—the tint of the erysipelas purple, or pale, then withhold the lancet, for God's sake, and exhibit the stimulants or tonics, which symptoms, if they be Nature's language, demand in a tone that should not be mistaken, and must not be contemned.

Dr. Macfarlane relates some cases of erysipelas. We think that, in a volume of clinical reports, more space might be usefully devoted to a subject of such paramount importance, and one on which disputes have run so high. Dr. Macfarlane, however, offers some compensation for his brevity, in the sensible remarks which he makes upon the nature and treatment of the malady. To those observations we shall draw attention first, and introduce some of the cases afterwards.

Dr. Macfarlane admits the natural distinction into cases in which inflammatory action predominates, and those in which the symptoms are indicative of want of power.

"It must be evident, from what has been already stated, that although the disease is always, strictly speaking, accompanied by inflammation, yet it is occasionally met with when, from the trifling degree of local excitement which exists, and the feeble state of the patient, antiphlogistic treatment is decidedly contra-indicated. To have recourse in such cases to local or general bleeding, purgatives or antimonials, would only tend to accelerate the progress of

---

* In Laputa, Gulliver informs us that the worst coats he ever saw were not cut by measure, but on abstract mathematical principles.

the inflammation, and to aggravate that peculiar condition of the system upon which the original development and propagation of the disease depend. Besides, the actual presence of inflammation does not always warrant us in adopting depletory measures. In scrofula, and particularly in some forms of strumous ophthalmia, although inflammation be decidedly present, we do not hesitate to prefer tonics and stimulants to antiphlogistic means; on the contrary, daily experience teaches us, that the latter class of remedies, if employed to the extent which the local inflammation seems to call for, will often produce the most injurious effects."

We repeat that the great rule is to study the individual case, and the best practical surgeon is he who can do so with most judgment and discrimination.

" I am ready to acknowledge, however," continues the reporter, "that in a great majority of cases, erysipelas is most effectually controlled by antiphlogistic treatment, such as venesection, local bleeding, by means of leeches, scarifications, &c.; emetics, purgatives, diaphoretics, &c. These means are generally resorted to in this hospital, and the affected parts are covered with a spirit lotion, unless there exists a good deal of vesication, when dry flour or fleece cotton is applied."

If Dr. Macfarlane has found venæsection required in the great majority of cases in Glasgow, we must say that we have not found it so in London. In the greater number of instances that have fallen under our observation, and the experience of a large hospital has shewn us not a few, the patients required neither bark nor bleeding, but did extremely well under simple treatment—occasional purgatives, diaphoretics, diluents, and light tonics on the subsidence of the complaint. An emetic in the first instance, and one or two doses of calomel to stimulate the action of the liver, are usually productive of advantage.

Dr. Macfarlane recommends spirit lotion as an ordinary application, but prefers dry flour or fleece cotton if much vesication exists. We conceive that some discrimination is necessary in selecting warm or cold applications. If the erysipelas is of an active kind, and the patient strong, cold spirit lotions are usually most grateful, if not most advantageous. But if the tint of the erysipelas is dull, the patient debilitated, and if a disposition to much shivering exists, warm fomentations appear, on every account to be preferable.

Dr. Macfarlane has not noticed the application of slight pressure in some cases of erysipelas, in which, along with a languid condition of the patient and a pale or purplish tint of the eruption, there is a disposition to œdematous swelling of the subcutaneous texture. The employment of strips of linen or calico, spread with a mixture of soap cerate and lead ointment, and gentle compression by means of a bandage over these, appear to be serviceable in obviating the occurrence of small collections of matter which are frequently found in cases of this character.

" In several cases of traumatic erysipelas, I have succeeded, after free vomiting and purging had been produced by the emeto-cathartic mixture, in arresting the progress of the disease, by rubbing the inflamed surface with lunar caustic. By this local stimulus, the morbid action of the part seems to be changed, its extension prevented, and in a few days the blackened cuticle is thrown off, without producing the slightest deformity. I have also attempted to circumscribe the disease when confined to the extremities, by encircling the limb a little way above the seat of the inflammation with a narrow stripe of blistering plaster. This was only successful when the erysipelatous affection was purely cutaneous, and not when the subjacent cellular substance appeared to be in the slightest degree involved."

The latter paragraph is quite in accordance with our own experience.—We have never seen benefit, though we think we have found mischief from the application of lunar caustic when the cellular tissue was affected. Even in the purely cutaneous erysipelas, which is very rare, we have seen the Rubicon, formed by the caustic, passed; whilst in those instances, in which the erysipelatous inflammation seemed hemmed in, we have not been persuaded that the patient did better than under ordinary circumstances. On the whole, we are not advocates for the use of lunar caustic.

"In phlegmonous erysipelas, I rarely have recourse to general blood-letting, and that only when the patient is young and robust, the pulse full and strong, or when, during the progress of the disease, the meninges of the brain become affected; the propriety of local bleeding, however, can hardly be disputed. For this purpose leeches are freely applied, and when the disease is in an incipient state, and the tumefaction and tension are inconsiderable, its progress may by such means be often arrested. I have only twice had an opportunity of adopting the practice of making numerous small punctures into the affected part, as recommended, and apparently so successfully employed, by Dr. Dobson of Greenwich Hospital.* In one of these cases, the affection was consequent on a wound of the scalp, and was decidedly benefited by this treatment; but in the other, which was an acute case of phlegmonous erysipelas of the leg, more than one hundred punctures were made with but trifling advantage. A considerable discharge of blood was obtained; but, when the punctures penetrated the cellular substance, in order to unload this texture of the effused serum, it was found that many of the openings became speedily closed by the protrusion of minute portions of adeps, by which the farther escape of fluid was prevented, and but little effect was produced on the tumefaction or tension of the limb.

The most powerful and decided method of checking this troublesome disease, is that by incisions into the affected parts,—these being carried through the integuments and cellular texture, and even through the fascia, should the disease appear to have penetrated so deeply. This practice has been almost uniformly adopted during the last ten or twelve years by the different surgeons who have officiated in this hospital, and, so far as I have learned, its results have been eminently successful, and have tended to confirm the favourable statements of Mr. Copland Hutchison,* who is justly entitled to the credit of having first introduced this powerful and efficient treatment. If incisions are employed at an early period of the disease, they remove tension, and, by diminishing vascular action, check the progress of inflammation, and prevent those diffuse and destructive suppurations, with sloughing of the cellular texture, which are so apt to ensue. Even at a more advanced period they are useful, by facilitating the discharge of purulent or sero-purulent fluid, and the separation of sloughs; and they also tent to limit the ulcerative destruction of the integuments. The diminution of the pain, redness, and swelling, is often strikingly visible after this practice has been adopted, and the relief of the constitutional symptoms is seldom less apparent."

We have tried Dr. Dobson's method in some cases, and have seen it tried in many. We do not entertain a very high opinion of it. In the majority of instances it did no good, and considering that it is painful, we think this a powerful objection. In fact, we believe that, in cases where the cellular membrane is little implicated, punctures are seldom necessary; and where it is severely affected they are useless. At the same time, there may be cases in which the discharge of serum from the distended cellular tissue may tend to prevent suppuration and sloughing.—We do not wish to proscribe the practice, but it has not been our lot to see much advantage from it.

In cases where there is considerable effusion in the subcutaneous cellular tissue, where the skin is intensely red, or where it is affected with a contrary condition, being purplish and inclined to slough, in such cases no practical

* Medico-Chirurgical Transactions, vol. xiv. p. 206.

* Medico-Chirurgical Transactions, vol. v, surgeon will hesitate for an instant to make bold incisions.

Dr. Macfarlane offers some remarks on the comparative advantages of large and small ones.

"I have attempted, in several cases, to contrast the practice of Mr. Hutchison, who makes a number of small incisions into the affected parts, with that of Mr. Lawrence, who recommends one or two long incisions to be made in a direct line through the middle of the inflamed surface. When the disease extends over an entire extremity, and the tension is uniformly diffused, and so great as to indicate an affection of the subfacial cellular texture, I have experienced more benefit from one or two long incisions, than from smaller and more numerous ones. But when the disease is exterior to the fascia, as most frequently happens, and there exists an unequal degree of swelling and tension in different parts of the affected surface, at some distance from, and not in a direct line with, each other, indicating the presence of gangrene or suppuration, in circumscribed portions of the cellular texture, one or two long incisions have not appeared to me to be so successful in either relieving the urgent symptoms, or checking the progress of the disease, as making a number of small cuts into those detached and distant parts of the inflamed extremity in which the swelling and tension predominate."

This is judicious advice, and we know not that we could usefully add to it. It is evident that on this, as on other occasions, the dispute has been carried to extremes, and that circumstances must regulate the length, as well as the situation of incisions. We must say that in general one free incision is better than two or three imperfect ones, but a cut of more than a certain length becomes of itself a serious complaint.—We repeat that the management of the individual case must be left to the discretion of the surgeon.

We shall now present some of the cases.

Case 1. *Erysipelas of the Face, treated by Stimulants and Tonics.*

"M. O., æt. forty-six, when admitted on the 6th May, 1826, had the right side of the face affected with an erysipelatous inflammation of a dull red colour, which had commenced three days previously on the right cheek, where the cuticle had been accidentally abraded. He was a dissipated and debilitated subject; his pulse was one hundred and twenty, and feeble; the tongue dry and furred; but the skin was cool, and the functions of the sensorium undisturbed. The affected parts were covered with flour, and, after opening the bowels by a mild purgative, two grains of the sulphate of quina were ordered every six hours in an ounce of wine.

On the 8th, the disease had extended over the scalp to the opposite side; the pulse was more rapid and feeble; the pupils contracted and irritable; the tongue typhoid; and there was hiccup, with low muttering delirium and drowsiness. The wine was increased, small doses of the carbonas ammoniæ prescribed, and the quinine continued. In five days the inflammation had ceased to spread; the cuticle desquamated; his strength improved; and he was dismissed on the 28th."

This case displays a feature which is not uncommon. A patient with erysipelas is apt to become low in a sudden manner. On one day the symptoms are scarcely alarming—on the next, they may wear the worst characters of typhus. It is the anticipation of this rapid depression that constitutes a great part of the surgeon's tact in the treatment of this disease. He must learn to distinguish when the pulse is beginning to falter, when the tint of the erysipelas is altering or waning, when the constitution is tired of its efforts. Unless he can do this he will probably lose many patients during his career. The following case may afford some illustration of these remarks.

Case 2. *Fatal Erysipelas.*

"M. F., æt. 8, had been in the Infirmary for several weeks, in consequence of an ulcer on the abdomen, between the pubes and umbilicus, which had nearly healed, when erysipelas commenced on the surrounding integuments, on the 1st of July, 1826. The disease extended rapidly over the abdomen, front and sides of the chest, neck, shoulders, and arms, and the affected parts were hot, painful, of a bright red colour, and, in some places vesicated. Vomiting and purging were produced by the emeto-cathartic mixture; a few leeches were applied, and afterwards a tepid spirit lotion. On the 2d, she was delirious; the inflammation was extending; the tongue was typhoid; the pulse was rapid and feeble; and the prostration of strength more decided. Wine, quina, and carbonas ammoniæ were ordered; and, during a continuance of the tonic treatment, the inflammation lost its vivid red colour, and ceased to extend. Her strength, however, decreased rapidly; vomiting and hiccup supervened; she became comatose, and died during the night of the 5th, with all the symptoms of typhus gravior.

The only morbid appearances discoverable on dissection, were superficial and deep-seated congestion of the brain, with a slight effusion of serum under the arachnoid.

We here see how rapidly prostration supervened—how feeble were the powers of medicine when it had occurred. Let not the young practitioner be rashly tempted into *active* antiphlogistic treatment in the early stage of erysipelas. He may repent it.

CASE 3. *Phlegmonous Erysipelas of the Arm—cured by Incisions.*

A. R., æt. 49; when admitted on the 3d of February, 1832, his left arm, fore arm, and hand, were greatly swollen, tense, and of a dark red colour, the disease having commenced six days previously without any known cause. The affected parts were hot and painful; there were several large vesications on the inner side of the fore arm, which contained milky serum and greenish-coloured lymph, and on the outer side, in one or two places, obscure fluctuation was perceptible. Four incisions were made through the skin and cellular membrane of the fore arm, (two of these were five inches, and the others two and a half inches in length,) and gave exit to a small quantity of pus. The divided integuments retracted considerably, and the tumid cellular substance, which had a red appearance, bulged out at the incisions. Immediate ease was obtained; the inflammatory appearances and constitutional excitement diminished rapidly; and when the bleeding had stopped, which amounted to twelve ounces, oiled lint was applied to the incisions, and the arm covered with a tepid spirit lotion. In a few days resinous ointment was substituted, and a bandage applied, under which the parts continued to heal slowly.

We have only room for allusion to another subject, erysipelas of the scalp.

"When erysipelas affects the head, the skin, and subjacent cellular texture are the parts generally affected, and the ordinary antiphlogistic means seldom fail in removing it. But when the disease is seated under the aponeurosis, diffuse suppuration and sloughing are speedily produced, unless prevented by the free and timeous use of incisions. I have seen more than once the neglect of this treatment prove fatal. During the Summer of 1826, a robust labourer was admitted into the Infirmary under my care, several days after an attack of phlegmonous erysipelas of the scalp had terminated in extensive suppuration. The head was enormously swollen; the scalp, which was ulcerated in various points, had a spongy or boggy feel, and was completely detached from the skull. Although incisions were employed to favour the escape of matter and the separation of sloughs, nearly the whole integuments of the head were destroyed by ulceration, and the patient died in a few days from effusion on the brain."

In a former number of this Journal we gave a very full report on erysipelas of the head and face, and on diffuse inflammation of the cellular tissue beneath the aponeurosis of the occipito-frontalis muscles. In that report will be found an attempt at pointing out the distinctive marks of this serious affection, and cases illustrative of the value of incisions. This is a subject of the utmost practical importance. We shall copy one case from Dr. Macfarlane.

*Case* 4. "A. C. æt. 38, received a small puncture-wound over the middle of the left parietal bone, on the 7th of February, 1827, and on the 10th he was admitted into the Infirmary. There was a slight blush of redness around the wound, which appeared to be suppurating, and the integuments of the left side of the head were greatly swollen, painful and œdematous. He complained acutely of pain and throbbing in the head; the pulse was 108, and strong; the tongue furred, but moist; the skin hot and dry. He was immediately bled to twenty ounces; twenty-four leeches were applied to the left side of the head, followed by a cold lotion; and free vomiting and purging was produced by the emeto-cathartic solution. On the 11th the disease had extended to the right side and forehead; the swelling and pain had greatly increased, and the general disturbance was aggravated. Notwithstanding a repetition of the leeching, with frequent doses of calomel and James's powder, the progress of the disease was not arrested. On the 12th, the whole scalp was involved, and the integuments, although little discoloured, were enormously swollen, and pitted on pressure.

As, besides the affection of the subcutaneous cellular tissue, there was also present a considerable tumefaction of the parts under the aponeurosis; and as the ordinary antiphlogistic means had failed in controlling the disease, I made six incisions into the scalp, in various points, each being about two inches in length, and carried through the aponeurosis. Sixteen ounces of blood, apparently arterial, were lost; the pain was immediately relieved, and the swelling diminished rapidly. For several days there was considerable discharge from the wounds, but no sloughing of the exposed cellular substance took place. He left the hospital, quite recovered, on the 2d of March."

In conclusion we may hint some deficiences in this report. The varieties of erysipelas might have been discriminated with more precision — the progress of the disease, and the characters requiring alterations in the treatment might have been more pointedly described—the distinction between the ordinary erysipelas and diffuse inflammation of the cellular tissue might have been an useful subject of allusion. We certainly think that so practical a surgeon as Dr. Macfarlane might have remedied these deficiencies even in a report. Yet still we are pleased to find that he has adopted such judicious views, and recommended such appropriate treatment.

---

### Birmingham Eye Infirmary.

Report for the Year 1833. By Mr. Middlemore, Assistant-Surgeon.*

The following numerical statement displays the relative frequency of various affections of the eye; amongst the poorer classes in Birmingham, and probably in most great towns.

" Simple acute conjunctivitis, 230. *(a)* Chronic coujunctivitis, 112. Acute conjunctivitis, with pustules on the conjunctiva, 123. Acute conjunctivitis, with pustule or ulcer of the cornea, 124. Acute conjunctivitis, with puriform secretion, 87. Purulent conjunctivitis of newly-born infants, 41. Irritable conjunctivitis, 44. Strumous conjunctivitis, 70. Pterygium, 7. Corneitis, 13. Vascularity of the cornea, 11. Opacity of the cornea, 149. Conical cornea, 5. *(b)* Staphyloma of the cornea, 12. Impaction of foreign bodies in the cornea, 17. Simple acute sclerotitis, 4. *(c)* Rheumatic sclerotitis, 11. Staphyloma of the sclerotica, 4. Morbid attennuation of the sclerotica, without distinctly-formed staphyloma, 3. Affections of the membrane of the aqueous humour, 14. Simple acute iritis, with or without ulcer or opacity of the cornea, onyx, or hypopion, 47. Chronic iritis, 9. Syphilitic iritis, 6. Strumous iritis, 7. Prolapse of the iris, 6.

* Provincial Transactions, Vol. II.

Fungus from the iris, 5. Vacillation of the iris, 5. Cataract, 17. *(d)* Dislocation of the lens, 15. Choroiditis, 7. Retinitis, 2. Glaucoma, 4. *(e)* Hydrophthalmia, 2. Suppuration of the eye-ball, 7. Atrophy of the eye-ball, 3. Fungoid, and various anomalous tumors of eye-ball, 4. Neuralgia of the eye-ball, 7. Oscillation of the eye-ball, 5. Amaurosis of various kinds and degrees, 100. Diseases of the lachrymal passages, 19. Epiphora, 14. Strabismus, 8. Tinea, 105. Lippitudo, 12. Hordeolum, 5. Ectropium, 3. Entropium, 6. Inflammation of the eye-lids, 16. Œdema of the eye-lids, 9. Ulceration of the eye-lids, 6. Ptosis, 4. Adhesion of the eye-lid of the globe, 3. *(f)* Tumours in the eye-lids, 27. Wound of the eye-ball and its appendages, 42."

### CHANGES IN THE TARSAL CARTILAGES.

Mr. Middlemore alludes to a fact which he has observed—that obstinate chronic inflammation of the conjunctiva occasionally depends on a morbid state of the tarsal cartilages. Surgeons should be familiar with the fact, in order to avoid the liability to error in forming their prognosis.

" Several obstinate cases of chronic inflammation of the conjunctiva, depended on a shrivelled, uneven, and irregularly ossified state of the tarsal cartilage, and, although not absolutely curable, were yet capable of considerable alleviation by appropriate treatment. In very old persons, changes in the figure, the size, the consistence, and other characters of the tarsal cartilage, frequently take place, and, among these alterations, the undue incurvation of its extremities, or a shrivelling of its texture, with the deposition of specks of ossific matter, are the most common; and, of course, any material irregularity of surface or change of figure it may undergo, will influence the surface and condition of the palpebral conjunctiva which covers it, and produce certain effects upon the eye, correspondent, in their extent, to the changes the conjunctiva may have sustained. In a case of tumour of the eyelid which I recently attempted to remove, I was surprised to find that it consisted of a mass of cartilaginous matter, deposited upon the cutaneous aspect of the tarsal cartilage; it was, in fact, nothing more than a great and irregular enlargement of that part."

### STAPHYLOMA.

Mr. Middlemore recommends an operation for staphyloma, which he has now practised for many years. It is only a slight modification of the practice recommended by Scarpa, Guthrie, and others for some forms of the disease, but Mr. Middlemore considers its applicability more extensive, than they would appear to have done. He relates a case in illustration of his opinion and advice.

*Case.* Sarah Mace, æt. 20, has a large spherical staphyloma of the left cornea, consequent on an attack of gonorrhœal ophthalmia. The diseased eye-ball is somewhat inflamed, and the opposite eye is in a very irritable state.

" Having placed her upon a table as for the operation of extraction, I introduced Beer's knife, at about an equal distance from the summit and base of the staphyloma, and brought out its point at a corresponding situation on the opposite side, and by gently urging the knife forward, a small semicircular flap was formed, which was immediately removed by the convex-bladed scissors, by an incision which constituted, as respects its outline, a portion of morbid cornea, of an equal size, and of the same form. The lids were then carefully closed, and a roller so applied as to keep them in apposition with the globe.

The operation occasioned very little pain; no bad symptom followed its performance, and in the course of a few days the opening in the cornea had closed, chiefly by the adhesion of its edges to a small portion of lymph in the centre. As this lymph became absorbed, the part diminished in magnitude, and, at the present time, the previously staphylomatous eye is smaller than the opposite and healthy globe, which is partly owing to the partial absorption of the morbid cornea, a circumstance which generally takes place when a portion has been removed from its centre for the cure of staphyloma."

Where the eye-ball is much enlarged, and the divided edges lie widely apart, it is better to bring them into contact, for the purpose of preventing the reproduction of the malady, of obviating the occurrence of acute inflammation of the exposed interior of the eye-ball, and, also, of diminishing the risk of staphyloma, which is apt to occur if the interior of the globe becomes filled with fresh secretion, before the matter of reparation possesses a sufficient degree of firmness to resist the pressure from within.

### Rheumatic Sclerotitis.

The real nature of this disease is often overlooked, and its progress is consequently unchecked. If the inflammation is obstinate, the pain intense, and somewhat periodical, the eye-ball not being particularly vascular, and the deep-seated textures being unaffected with evident acute inflammation, Mr. Middlemore is in the habit of employing the following treatment, with success.

An active cathartic is first prescribed, and, afterwards, a few grains of calomel and Dover's powder, to be taken every night at bed time, and three or four grains of quinine aboutthrice daily. A small quantity of the strong mercurial ointment, blended with a grain of opium, is directed to be rubbed above the eye-brow, about the situation of the supra-orbitary nerve, every evening, if the pain in the eye-ball and orbit is intensely severe. If this plan fails to afford much relief, half a drachm of the wine of colchicum is directed to be taken thrice daily, and two pills are given in the evening, consisting of five grains of blue-pill, and six of the extract of conium. By these means, with the aid of fomentations, Mr. Middlemore is seldom foiled in his treatment of the complaint.

### Dislocation of the Lens.

Passing over some remarks of Mr. Middlemore's, we may state that it is his object to point out the general mode of practice adapted for the relief of the more common cases of this affection.

An adult patient, perhaps, presents himself at the Infirmary, who, after sustaining a severe fall upon the head or concussion of the body, or after some violent straining effort, may have perceived a dimness of vision,* and uneasiness and inflammation of the eye-ball. On carefully examining such an eye, it will, very probably, be found that the lens is slightly opaque, that it is pressed, but not forcibly, against the iris; and that there is some degree of external ophthalmia, with a slight zonular arrangement of vessels around the cornea. In such a case, different surgeons would pursue different methods of treatment. Some would directly remove the lens—others would merely resort to measures for the relief of the inflammation—and others would diminish the inflammation first, and then extract or break up the lens. For reasons into which we need not enter, and many of which will be obvious to those acquainted with ophthalmic surgery, Mr. Middlemore recommends the last-mentioned mode of treatment—the subduing of the immediate acute symptoms, and the subsequent removal of the lens, if it has not been absorbed, and if it constitutes an adequate personal deformity, or continues to augment the severity or prolong the existence of inflammation.

If the dislocation of the lens is associated with a blow on, or wound of, the eye, then the nature of that injury must be taken into consideration. Mr. M. believes that, as a general rule, it would be useless and injurious, in such circumstances, to attempt the extraction of the lens. The dislocation of the lens, and the inflamed condition of

* "The dimness of vision may, of course, depend on *concussion of the retina*, and may not be owing to the displacement of the crystalline."

the eye, are excited by the same cause, the violence of the injury inflicted.

"There is one other variety of dislocation of the lens, to which I shall, for a short time, direct attention; and it will afford me great pleasure if my brief allusion to it, should induce surgeons of greater experience, to favour the profession with their opinion, of a condition of disease which is sometimes adequate, to render the subject of it a burdensome and comparatively useless member of society.

A blow upon the eye may displace the lens and its capsule, and, at the same time, rupture the hyaloid membrane; or it may inflict such injury upon these parts, that the septa of the hyaloid membrane may become absorbed, and the vitreous humour acquire a morbid fluidity, so that the lens will sink backwards and rest upon the retina, and, by its compressive effect, cause total amaurosis. Now, in such instances, an examination of the eye, with the head inclined backwards, will detect a vacillating iris, and a slightly green-coloured body (which is the most common change of colour that the lens undergoes under such circumstances), at the lower and posterior part of the globe; and, whilst in this position, the vision of such an eye will be entirely destroyed. If the patient move the eye-ball freely and rapidly, the lens, or a greenish or amber-coloured body, will be seen to roll about in the eye. If the patient lean the head forward, the lens will rise into its proper place, or, perhaps its upper margin may be seen, if the pupil is somewhat large, and he will be able to see in this position, not perfectly, but sufficiently well to find his way about, even in walking in a strange road, or in a place to which he has not been accustomed. Now it may happen that, in some of these cases, the lens may not be wholly detached, a slight connexion still existing at its lower margin, and then, of course, it will not float about so freely in the vitreous humour, and will more readily come forwards if the head be inclined in such a direction; in other instances, the lens and its capsule appear to be wholly detached from the hyaloid membrane, and, of course, when this takes place, the crystalline body will, in the erect position of the body, sink downwards, as an effect of its weight, and, by resting upon the retina, induce complete amaurosis.

I have seen many of these cases recently, and shall briefly refer to one or two of them, but, I must confess, I had never noticed this form of injury, until my worthy and enlightened friend, Mr. Smith, of Southam, obligingly sent to me a man, in whom the lens passed freely into either chamber of the eye, in accordance with the position of the head, sometimes coming in front of the iris and pressing that membrane (which was in a state of vacillation), towards the retina, and on leaning the head backwards, it would pass the pupil and resume its proper position, so that, I apprehend, in this instance, the hyaloid membrane was entire. It may be desirable to state that the lens preserved its transparency."

Mr. Middlemore mentions two other cases, which, however, we need not introduce. He observes, with respect to the treatment, that the lens cannot be broken up or destroyed by any means short of extraction. This he thinks it only desirable to perform where the sight of the other eye is seriously impaired, or the affected one is suffering a great degree of irritation from the agitation of the lens, or much interruption to vision from its movements.

"In the instances to which I have referred, I have operated in the following manner. The patient being seated in a chair, with the head a little inclined forward, I make a free incision at the lower part of the cornea, with the triangular extraction knife, and having introduced a small hook beneath the flap, I succeeded in drawing the lens through the opening; in one case, after considerable delay, in the other, without the slightest difficulty. The difficulty in removing the lens, arose from its great mobility, and the impossibility of fixing the hook in the central and more solid part of its substance."

### On the Occasional Effects of Tumors in the Eyelids.

Tumors in the eye-lids, and especially when near the tarsal margin, or situated between the tarsal cartilage and the palpebral conjunctiva, or when arising from the latter part, or growing from the precise edge of the eye-lid, will sometimes give rise to acute or chronic ophthalmia, and also excite amaurotic symptoms. Mr. Middlemore frequently removes tumors from the eye-lids, for the cure of acute or chronic ophthalmia, which had resisted every variety of previous treatment, but which yields with amazing rapidity to this. He relates three cases of this kind, which deserve attention.

"A child was sent to me, a few days ago, with acute ophthalmia, which, to copy the words of its medical attendant, had refused to yield to every method of treatment he could suggest; it was also mentioned, that a little tumour existed in the eye-lid; but as it was very small, at some distance from the tarsal margin, and inadequate, as was presumed, both from its size and situation, to give rise to any irregularity of the mucous surface, it was considered to have no influence in maintaining the inflamed state of the eye. On carefully examining this child's eye, I found it to be acutely inflamed; and I discovered, also, a minute tumour situate between the tarsal cartilage and the conjunctiva lining the eye-lid; and although I perceived the propriety of removing this tumour, yet, as the eye was a good deal inflamed, I certainly did not consider this to be a favourable time for its extirpation; I therefore recommended various measures which usually relieve conjunctivitis, but without any beneficial result; and I then removed the tumour, at the child's second visit, and with the effect of curing the ophthalmia in a few days, without the use of any lotions or medicines of any kind.

A gentleman had a small granular excrescence at the margin of the upper tarsus, with great dimness of vision of the eye of the same side. I removed this warty growth with a fine ligature; and which eventually removed the amaurosis also, an effect by no means expected or contemplated by the patient.

Miss A. of Birmingham Heath, had a small fungoid growth at the edge of the left inferior tarsus, with various impairment of vision of the left eye. This growth was almost entirely removed by the application of the sulphate of copper: and her sight was completely restored. However, I ought to mention, that she took, at my recommendation, the carbonate of iron during the time she was in attendance at my house, for the purpose of having the sulphate of copper applied to the tumour."

This concludes the report of Mr. Middlemore. He appears to be zealous, industrious, and intelligent; qualities calculated to ensure his success. We trust he will continue to detail the results of his private and public experience.

---

### Middlesex Hospital.

#### I. Wounds of the Knee-Joint.*

Sir Charles Bell, in a clinical lecture on Injuries of the Knee-joint, published in the Medical Gazette, refers to some cases which have lately occurred in the institution to which he is attached.

*Case* 1. A boy, 14 years of age, was admitted under the care of Mr. Arnott, with a penetrating wound of the joint. He was a shoemaker; and while he was at work, a sharp knife which he was using slipped, and ran into the knee, about an inch deep; it entered at the inner part of the knee-joint, just above the patella. On admission, a small wound only was observed; but the knee was inflamed, and attended with considerable effusion; there was also pain upon the slightest motion. Leeches and ice were applied, and the wound was covered with plaister and a solution of sealing-

* Med. Gazette, May 17th, 1834.

wax. After this, no bad symptom was observed; at least such was the case seven days after the injury, the period when Sir Charles Bell's lecture was delivered.

The able lecturer observes, that this is "a pleasant case to set out with," adhesion of the outward wound removing apprehension. The next case displays the series of evil consequences which is apt to succeed a wound of the articulation. It may be told in the language of the lecturer.

"There is another case, that of Cooper in Clayton's ward. 'He is 18 years of age, and has had a severe wound of the left knee-joint; it was occasioned by his falling upon his scythe. He is a strong lad. The point entered the knee-joint, penetrated the capsule, and, it is supposed, cut the cartilage. The inflammation is already considerable. Leeches have been applied, and cold lotions; but notwithstanding all the means employed, the inflammation continues unabated.' The next report says, that 'the inflammation involves the whole leg and thigh.' Another report is dated some weeks afterwards; it states that 'abscesses form in succession in various parts of the thigh and leg, as well as in the joint, and that he is now reduced extremely low, being very faint, so that poor hopes are entertained of his recovery.' It appears, that after remaining in this state, in danger of dying from hectic, with night sweats and diarrhœa alternating, the constitution of a fine healthy country lad struggling with the disease, he seemed to rally a little with the assistance of generous diet, wine, porter, and all that could be given him to support his strength. At length slowly and progressively he shewed improvement; the great swelling of the limb subsided, the abscesses closed, and there was a hope of his being dismissed with an anchylosed joint. But then came another mischief, which all are subject to in large hospitals, a relapse, attended with erysipelas involving the leg and thigh, and having a most prejudicial effect on his constitution, and carrying him back we may say three months in the cure. He again recovered; and after being a patient seven months, he was discharged with a stiff-joint."

This case affords occasion to Sir Charles to point out the effects to be dreaded when the wound does not unite. Suppuration in the joint—extension of inflammation and suppuration to the veins of intermuscular cellular tissue external to it. On these mischievous results the lecturer makes some eloquent remarks. From wounds of the articulation he slides by an easy process to the subject of loose cartilages. He appears to dread the operation, and deprecates it strongly unless the disease is productive of most serious inconvenience. He mentions the case of a young surgeon who was absolutely precluded from successfully pursuing his profession. When he was called to a patient, and at the moment when he was about to cross the saddle, the loose cartilage would frequently get between the heads of the femur and the tibia, when he required to be lifted from his horse, to be placed in bed, and to remain there for some days.

Under ordinary circumstances the best plan is to apply an elastic bandage and compress, which will cause the absorption of the effused synovia which occupies the joint. The bandage confines the loose cartilage in its recess, and prevents its getting between the ends of the bones, which it is most apt to do when the capsule is distended with fluid.

Sir C. Bell relates an interesting case which occurred in the hospital.

"A woman was admitted with a loose cartilage in the joint. She stated that, 'she began to feel her knee painful, and something move in it, about three years ago.' She said, 'she obtained no rest day or night, and there was no cessation of the pain; she could walk, but she was subject to occasional extraordinary lameness;' and, in short, 'that her suffering was so great that she was willing to undergo any hazard through an operation, with the hopes of relief.' The operation was performed; an incision was made on the inside of the patella; two loose cartilages were withdrawn; severe inflammation very soon followed, with frequent rigors and constitutional disturbance. All the means for subduing inflammation and pain were had recourse to with every possible attention, but the wound did not adhere; inflammation and suppuration commenced in the joint, and extended to the leg and thigh, very much in the manner we have seen it take place in the cases of accidental wounds. There followed the inflammation and suppuration, that exhausting hectic of which I spoke; then came sloughing on the hip—and what does this sloughing of the integuments of the sacrum imply, if it be not that, owing to the continued suffering and exhaustion, the patient is kept to one posture by the excess of pain? There were no hopes of saving her life, except by amputation; the operation was performed and from that time she began to rally, and she left the hospital quite well."

Sir Charles Bell observes, that the surgeon must go to the operation with fear and trembling. Were it not that there is at present too great a rage for operating, we should almost imagine that an injunction of this kind coming from so high a quarter, must depress the spirits of the young operator, and unnerve his hand when it most required steadiness. But the able lecturer is one whose cautions are dictated by judgement, and who alarms to save.

The following is his method of performing the operation.

"I do not think I could perform the operation neatly, so as to please myself, where there were two cartilages to remove; the reason of which you will perceive if I describe the method. First, you must teach your assistant what he has to do; for the success of the operation is fully as much dependent on the assistant as on the operator. I have a piece of board cut, with a semicircular notch; and by means of this, having got the foreign body on the lateral part of the knee, just on the side of the lower head of the femur, I force it up to the angle where the synovial membrane is reflected. I push the board with the notch firmly against the loose cartilage, holding it, as it were, in a corner at the utmost point of the cavity of the knee. Placing this in the hands of the assistant, he must keep it steadily in that position, for if he does not, the cartilage will escape; and if it escape, misery follows. The next thing that you have to consider is, that you are not to cut directly down, because you have to provide for an oblique wound, that the lips may more certainly heal, and therefore you draw the integuments a little aside; and having done that, you then cut down on the cartilage. I say, then, having drawn the integuments aside, you carry your knife (sharp as a razor) across, very lightly, and cut with a perpendicular edge, taking care to avoid pressure; for if you press on the cartilage, it may escape. At last you see nothing but the cobweb of the proper capsule; you continue to draw the knife over it, and out starts the loose cartilage. But sometimes I have done thus: having made the incision through the integuments, I take a strong and straight couching-needle, and strike it through the thin membrane, direct on the cartilage, and so hold it firm; and then, drawing the knife by the side of the point of the needle, I bring out the cartilage on the point of the needle, which makes a pretty operation. If the body escapes after you have made the incision, and the synovia has come out, and you have to bend the knee to get hold of the cartilage, or if you have to put in a pair of forceps to catch it, or having adroitly brought out one, there is a second, as there was in this case, it is ten chances to one that you cannot remove it with so slight and oblique an incision as I have described; and then all the mischief that I have dwelt upon is too apt to result. You would hardly believe that a cut made with so clean an instrument should produce this mischief, yet the well-informed student knows that it must be so, because, although the operation be done according to rule, and is one of the admitted or regular operations, still it is a penetrating wound; and if adhesion does not follow, misery and suffering result—the loss of the limb or the loss of life."

Sir Charles Bell alludes to a case of rupture of the internal lateral ligament, and, in his characteristic style, he adverts to the frequency of relaxation of this ligament, especially in elderly females. It usually occurs from the individual making a false step, the obliquity of the femur causes the weight to bear on the internal lateral ligament, it is stretched, the obliquity of the thigh-bone is increased, and repetitions of the accident are frequent from this circumstance. At last the patient cannot step at all.

"The obliquity of the bone, and the weakness of the ligament, are such, that she cannot bear her weight upon the limb. If, then, you are sent for to an old lady, and she tells you that coming out of the carriage she sprained her knee, you know that it is most likely the inner ligament which is hurt; and in examining the knee, you put your finger or thumb on that ligament, and ask whether the pain is not there. She replies, 'it is,' and it shews her at once that you are acquainted with the case."

The treatment consists in the removal of inflammation in the first instance, and in the subsequent application of an appropriate mechanical contrivance to support the limb.

### II. ERYSIPELAS.

As a pendent to the article on erysipelas founded on the cases of Dr. Macfarlane, we may introduce some observations delivered by Mr. Arnott in a clinical lecture at this hospital. That gentleman relates eight cases, and concludes with an exposé of his opinions.

"I stated to you, gentlemen," says he, "that a difference of opinion still prevailed as to the nature of erysipelas, and of its treatment. Now with regard to the former, let us first consider what has been the character of the phenomena presented to you in the preceding cases. You have seen, then, in all, and in the majority after injury, redness, swelling, heat, and pain of the common integuments, accompanied by more or less of general febrile disturbance. To the local affection you cannot hesitate to apply the name of inflammation. It has, indeed, been said that it is not a pure, a healthy inflammation, and chiefly from the different course it observes from a phlegmon—the essential difference being its great tendency to spread. Now, without alluding to the impropriety of the term *healthy* inflammation being applied to any particular form of the disease, seeing that they are all morbid processes, you will at once perceive that the distinction does not hold good when you carry your observations to other parts of the body. In other membranes as well as the skin, inflammation, when once excited, readily extends. This is the case with the serous membranes, the mucous and synovial membranes, &c.; so that if a distinction is to be established on this ground, the majority of those which we meet with in practice would not rank under the head of healthy inflammations. It has been stated that there must be something peculiar in the inflammation of the skin in erysipelas, as it only occurs in some individuals: no doubt it is so, but there is nothing singular in this. Wounds, and very severe wounds too, of other textures, are not invariably followed by inflammation. In the instance, too, of internal inflammations, as of pleurisy or bronchitis, although many individuals are exposed to the supposed and less manifest causes, only a certain number are affected."

We may venture to hint, that these remarks of Mr. Arnott go to prove that there is no material difference between erysipelas and phlegmon. They tend to establish this, if they tend to shew any thing at all. Mr. Arnott's analogy will, however, admit of some dispute. There is no peculiarity, says he, in the circumstance of erysipelas being a spreading inflammation, because inflammation extends in other tissues, the serous, the mucous, the synovial. But suppose that in those tissues two modes of inflammation were observed—that in one there was a marked disposition for limited action; and in another little or no tendency to limitation—suppose, in short, that those tissues displayed, as the skin does, two kinds of inflammation—what would Mr. Arnott say then? We may be told that we beg the question—that we assume the existence of two modes of inflammation in the skin and the cellular tissue. Without arguing this point at any length, we may observe that the phenomena of phlegmon and of erysipelas are in many particulars opposed to each other, and that few practical surgeons will contest the fact. If phlegmon, then, be taken as a standard of inflammatory action, erysipelas is either something else, or differs from it in many important respects. To contend that it is, or is not inflammation, is a war of words, for probably the disputants are not agreed in their definition of the term.

These idle disputes are well calculated to display the futility, or, at all events, the weakness of system. Leaving to the schools such arguments, the practical surgeon observes the phenomena of disease, and endeavours to apply to the conditions he discriminates remedies suited to each. He cares not what erysipelas may be called, but, studying individual cases, he ascertains its laws, and endeavours to control its action.

We will not go over the ground we have already trod, in noticing the cases of Dr. Macfarlane. We need not repeat, nor even enlarge on, the observations we offered with respect to the varieties and the treatment of the disease. We propose, at an early opportunity, to lay before our readers a series of cases, illustrative of the various features of the malady, and calculated to exhibit the influence of treatment. We shall now content ourselves with recording the opinions and experience of Mr. Arnott on the latter subject.

"You would observe, then," says that intelligent surgeon, "that in all the cases which have been related the treatment was essentially antiphlogistic; the patients were, with one exception, placed upon low diet, had aperient medicine and mild antimonials. That, in some, general blood-letting was employed; in others, local; whilst in one-half neither the one or the other was deemed requisite:—that cold local applications were used in most instances; in others, warm fomentations:—that, in only one case, stimulants were tried, and were obliged to be abandoned immediately, from the aggravation of the symptoms which ensued.

In the employment of general blood-letting I am influenced by the condition of the patient, the seat of the affection, and the degree of the constitutional disturbance: where the patient is young and strong,—where the head and face are affected, or where there is considerable fever and a strong pulse,—it is always a question with me, whether I should not take blood from the arm. In one case (the 7th), a severe one of the head and face, where the patient's pulse did not authorize general blood-letting, leeches were applied to the temples, and repeated with marked relief. Aperients or purgatives are almost invariably useful; and where there is a foul state of stomach, I associate with them calomel or tartarized antimony. Respecting local applications, without making it an absolute rule, I generally order the cold or spirit wash, where erysipelas is the result of local injury. When the inflammation extends over the greater part of a limb, warm fomentations usually prove more agreeable to the patient's feelings than cold ones; and the same is sometimes experienced in erysipelas of the head and face of so called spontaneous origin. You have seen the progress of the inflammation of the skin in one case limited by the application of the nitrate of silver; and in another of phlegmonous erysipelas you have witnessed the decided advantages of incision. It is almost superfluous to observe, that in some cases, neither blood-letting, purgatives, or medicine of any kind, is requisite, but that simply by attention to diet and regimen, the disease will run its course in safety.

I am not unaware that a very contrary plan of treatment is adopted by some, that even to the young and strong wine and bark are given. Upon this I can only remark, that I have only very rarely found them either requisite or beneficial. That I do not entirely prohibit them, you must have noticed; but where they are employed, their effects should be closely watched, or in some cases irremediable mischief will be the consequence.

In conclusion, it may be as well for you to recollect, that in the treatment of the medical erysipelas at least you are rather to have in view the guiding your patient safely through the attack than the cutting of it short; and this object, I believe, you will more securely attain by adopting the general principles of treatment which you have witnessed than by any other."

There are two points on which we may lightly comment. Mr. Arnott states that in only one case were stimulants tried, and that then they occasioned an aggravation of the symptoms. Of course this is a fact, and cannot be disputed. But if we are to draw the inference that such is universally the case in the hospital to which Mr. Arnott belongs, we can only observe that stimulants are often found necessary for erysipelas in other institutions. The other remark we have to make is this. Mr. Arnott states that in the "medical erysipelas," (erysipelas of the head and face independent of injury,) the surgeon should rather have in view the guiding his patient safely through the attack than the cutting it short. And why should he not pursue the same object in treating the surgical erysipelas too? Does Mr. Arnott seriously believe that they are different in nature?

